# Integrated multiomics characterization reveals cuproptosis-related hub genes for predicting the prognosis and clinical efficacy of ovarian cancer

**DOI:** 10.3389/fimmu.2024.1452294

**Published:** 2024-11-12

**Authors:** Yang Xiaorong, Xu Lu, Xu Fangyue, Xu Chao, Gao Jun, Wen Qiang

**Affiliations:** ^1^ Department of Gynecologic Oncology, Jiangxi Cancer Hospital, The Second Affiliated Hospital of Nanchang Medical College, Jiangxi Clinical Research Center for Cancer, Nanchang, China; ^2^ Department of Integrated Chinese and Western Medicine, Zhejiang Cancer Hospital, Hangzhou, China; ^3^ The Second Clinical Medical College, Zhejiang Chinese Medical University, Hangzhou, China; ^4^ Department of Gynecologic Oncology, Zhejiang Cancer Hospital, Hangzhou, China

**Keywords:** cuproptosis, RiskScore, ovarian cancer, single-cell sequencing, bulk RNA sequencing

## Abstract

**Background:**

As a prevalent malignancy in women, ovarian cancer (OC) presents a challenge in clinical practice because of its poor prognosis and poor therapeutic efficacy. The mechanism by which cuproptosis activity is accompanied by immune infiltration in OC remains unknown. Here, we investigated cuproptosis-related OC subtypes and relevant immune landscapes to develop a risk score (RS) model for survival prediction.

**Methods:**

Cuproptosis-related genes (CRGs) were identified to construct molecular subtypes via an unsupervised clustering algorithm based on the expression profiles of survival-related CRGs in the GEO database. Single-cell datasets were used to estimate immune infiltration among subtypes. The RS oriented from molecular subtypes was developed via LASSO Cox regression in the TCGA OC dataset and independently validated in the GEO and TCGA datasets. Hub markers from RS were identified in tissues and cell lines. The function of the key gene from RS was identified *in vitro*.

**Results:**

We investigated cuproptosis activity and immune infiltration to establish three clinical subtypes of OC based the differentially expressed genes (DEGs) from CRGs to create an RS model validated for clinical efficacy and prognosis. Six hub genes from the RS served as ongenic markers in OC tissues and cell lines. The function of GAS1 in the RS model revealed that it exerts oncogenic effects.

**Conclusions:**

Our study provides a novel RS model including 6 hub genes associated with cuproptosis and immune infiltration to predict OC prognosis as well as clinical efficacy.

## Graphical Abstract

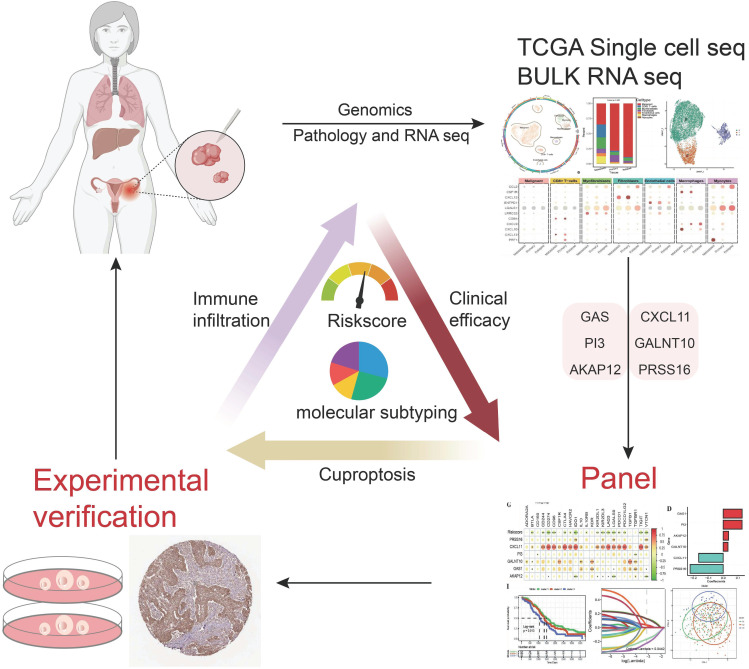

## 1 Introduction

As the leading cause of cancer death in the female reproductive system, OC is defined as a “silent killer” because of its insidious symptoms at an early stage and advanced disease at the time of diagnosis. According to the World Health Organization, there were approximately 313,959 new cases worldwide and approximately 207,252 deaths due to OC in 2022 (1). Given the high morbidity and mortality of OC, current diagnostic tools, such as the International Federation of Gynecology and Obstetrics (FIGO) stage system (2) and several common serum biomarkers, such as carbohydrate antigen 125 (CA125) (3) and human epididymis protein 4 (HE4) (4), are far from ideal models for precisely estimating the prognosis and curative effect of each patient. A reliable prognostic model is needed to accurately evaluate the prognosis of patients, which is crucial for optimal individualized management and treatment.

The tumor microenvironment (TME) is a highly complex and heterogeneous ecosystem consisting of tumor cells, infiltrating immune cells, and stromal cells intertwined with noncellular components, in which immune‐related genes and immune infiltrating cells play indispensable roles (5). Despite its high morbidity and mortality, OC is a recognized immunogenic tumor, and immunotherapies have attracted substantial attention because of their promising potential in OC therapy. However, cancer immunotherapy is not effective for everyone, and distinct immune cell infiltration patterns result in different responses to cancer immunotherapies (6). In addition, the variability in therapy response and the determinants underpinning these tumor immune phenotypes remain elusive (7). Thus, there is an urgent need to discover key molecular determinants involved in immune infiltration signatures and construct prognostic signatures based on these signatures.

Recently, a novel form of cell death pathway triggered by copper, named “cuproptosis”, which differs from apoptosis, pyroptosis, necroptosis, and ferroptosis, was discovered by Peter Tsvetkov and colleagues in 2022 (8). Copper is an extremely essential element involved in all types of biological procedures in the human body, especially in tumor growth and metastasis (9). Additionally, cuproptosis-related genes (CRGs) have been reported to be associated with immune cell infiltration in melanoma (10), esophageal carcinoma (11), and hepatocellular carcinoma (12), with an increase in protumor or antitumor immune components in tumors; however, few CRGs have been reported in OC (9). Due to the promising future of immunotherapy in the treatment of OC and the crucial role of cuproptosis in immune cell infiltration, evaluating cuproptosis may be an effective way to predict the prognosis and therapeutic benefit for patients.

In this study, molecular subtypes and prognostic models of CRGs in OC were established, and their relationships with the characteristics of immune infiltrating cells in OC were elucidated at the single-cell level. The results were further validated using four cohorts from major public databases. In addition, we aimed to illustrate the potential of the risk model to predict the efficacy of immunotherapy and chemotherapy. Our study revealed a potential association between cuproptosis, prognosis, and the TME in OC patients. These findings may provide a new method to predict outcomes in OC patients and ameliorate them.

## 2 Materials and methods

### 2.1 Data download and preprocessing

The FPKM expression profile data of OCs in the TCGA database were downloaded via the R package (TCGAbiolinks), and log2(FPKM+1) transformations were performed to calculate their abundance. Corrected TCGA survival data (13) were used for prognostic survival analysis without samples whose survival time was less than 30 days. Mutation data were also downloaded for genomic variation analysis. The clinical information of the patients in the TCGA_OC cohort used for the analysis is presented in **Table 1**. The expression and metadata files from the GSE130000 dataset were downloaded from the GEO database (https://www.ncbi.nlm.nih.gov/geo/) as the input files to construct Seurat single-cell objects for the analysis of the dataset. The expression and survival data of the GSE26712 dataset were downloaded from the GEO database as the GEO external validation set for the risk model. Moreover, the expression and clinical annotation data of the uroepithelial carcinoma dataset IMvigor210 were downloaded from http://research-pub.gene.com/IMvigor210CoreBiologies as the immunotherapy dataset without samples whose survival time was less than 30 days. Sample information for each of the above external datasets is shown in **Table 2**.

**Table 1:** TCGA OC cohort sample clinical information sheet.

TCGA cohort	Information	Number
**Status**	Alive	131
	Dead	211
**Age**	Age>=55	221
	Age<55	121
**Grade**	G1/2	41
	G3/4	292
**Stage**	Stage I/II	19
	Stage III/IV	320
**venous_invasion**	YES	60
	NO	32
**lymphatic_invasion**	YES	92
	NO	40

**Table 2:** Sample information table for GEO dataset.

Cohort	Subgroup Category	Information	Number
**GSE26712**	**Status**	Alive	56
		Dead	129
**IMvigor210**	**Response**	CR/PR	68
		SD/PD	227
	**Status**	Alive	108
		Dead	187

### 2.2 Gene set acquisition

The HALLMARK and GO gene sets were obtained from the MsigDB database (**Table 3**), as were the immune function-related gene sets from a previous article (14) (**Supplementary Table 1**: Immune_function_geneset). A total of 43 copper death-related genes were also obtained from previous work (15) (**Supplementary Table 1**: cuproptosis_related_gene).

**Table 3:** Table for immune infiltration.

Cohort	Information
**GSE130000**	**immune cell infiltration**
**MsigDB**	**immune function**

### 2.3 Differential expression analysis

Molecular subtypes of the TCGA_OC cohort were analyzed for differential expression via the limma R package, and differentially expressed genes (DEGs) were subsequently screened with Benjamini−Hochberg (FDR)-corrected thresholds of p values<0.01 and |log2FC|> 0.5.

### 2.4 Single cell identification and profiling

The single-cell dataset was analyzed via the specialized single-cell transcriptome analysis tool R package Seurat, which includes the steps of constructing objects, data normalization, data downscaling and clustering, identifying marker genes, and visualizing the analysis results. First, the single-cell sequencing results were selected for data merging, and the Seurat object was then constructed via CreateSeuratObject() of Seurat. After harmony, the data were further downscaled via uniform manifold approximation and projection (UMAP) to classify the cell types in the low-dimensional space. The cell subpopulations were identified via FindClusters(), and all the marker genes of all the subpopulations were identified via the FindAllMarkers() function. The function DimPlot() was used to visualize the results of dimensionality reduction of single-cell expression data and the distribution of active cell populations. Moreover, the R package plot1 cell was used for cell type proportion analysis and visualization of marker gene expression.

### 2.5 Single cell annotation

The R package SingleR was used to annotate the subpopulation results on the basis of Seurat 0.6 resolution, and BlueprintEncodeData were selected as the cell type reference database.

### 2.6 Identification of cuproptosis-active cell populations

The cuproptosis-associated marker genes of our malignancy cell subpopulation were used to calculate the activity score of each malignant cell via the R package AUCell and to determine the threshold for delineating active cells in the current gene set via the AUCell_exploreThresholds() function. The cell clustering UMAP embedding was then colored based on the AUC score of each cell to show which subpopulation-specific cuproptosis-associated factors were active in which cell subpopulations.

### 2.7 Construction of the prognostic risk model

One-way Cox analysis was first performed to screen the genes related to prognosis for intersubtype differences, and a prognostic risk score model for ovarian cancer was then constructed on the basis of this gene via least absolute shrinkage and selection operator (lasso). The Tibshirani (1996) method was used to screen the variables to reduce the number of genes in the risk model. The final multifactorial Cox regression model was established to construct the OC risk score (Riskscore) model.

### 2.8 Survival curves of gene expression

The TCGA OC tumor samples were divided into high- and low-expression groups, with the median gene expression as the threshold point. Survival curves for prognostic analysis were generated via the Kaplan−Meier method, and the significance of the differences was determined via the log-rank test.

### 2.9 Estimation of the proportion of immune infiltrating cells and the immune score

Based on the expression profile of the TCGA_OC dataset, four algorithms, CIBERSORT, TIMER, ssGSEA, and ESTIMATE, of the R package IOBR were applied to calculate the proportion of infiltrating immune cells.

### 2.10 Gene set enrichment analysis

Gene set (immune function gene set/HALLMARK) enrichment scores were calculated for each cancer sample on the basis of gene expression in TCGA OC samples via the ssGSEA algorithm of the R package GSVA, which first performs a kernel estimate of the cumulative distribution density function for each gene in all samples. The enrichment score differences between subgroups were then calculated via statistical tests, and the enrichment score heatmap was plotted via the R package pheatmap combined with the clinical characteristics of the samples. The correlations among the expression of model genes, risk scores and enrichment scores were also calculated via the cor() function and visualized via the R package corrplot.

Gene set enrichment analysis (GSEA) uses a predefined set of genes to rank genes according to their differential expression in two types of samples and then tests whether the predefined set of genes is enriched at the top or bottom of this ranking table. Enrichment analysis was performed through the R package clusterProfiler on the basis of GO functional gene sets and KEGG pathways, and the top 8 gene sets with significant enrichment results were then selected to generate bubble plots showing the enrichment results.

### 2.11 Genomic SNV analysis

Based on the maf file of somatic mutation detection results of the TCGA_OC cohort, the oncoplot() function of the R package mafTools was used to draw waterfall plots to show the differences in SNV mutations between different model groupings. Finally, the maf data of the high- and low-risk groups were analyzed via the mafCompare() function to obtain genes with significant differences in mutations between the two groups.

### 2.12 Immunotherapy response prediction

The algorithm is based on expression profiles prior to tumor treatment and scores multiple published transcriptomic biomarkers to predict patients’ immunotherapeutic response. The TIDE score (http://tide.dfci.harvard.edu) integrates T-cell dysfunction and exclusion features, simulates tumor immune escape with different levels of tumor-infiltrating cytotoxic T cells, and appears to be highly advantageous compared with other biomarkers. The immunophenoscore (IPS) can be used to identify immunogenicity and predict the response to immunotherapy in multiple tumor types. We obtained IPS scores for tumor samples in the TCGA_OC dataset via an online website (https://tcia.at/home) and performed between-group difference analysis via statistical tests.

### 2.13 Samples and cell collection

All tumor tissues were obtained from Jiangxi Cancer Hospital and stored in liquid nitrogen at -80°C until use. This research was approved by the ethics committee of Jiangxi Cancer Hospital (Approval number: 2022ky305). Every patient provided informed consent prior to the collection and usage of these clinical materials. The OC cell lines used in this study were obtained from the ATCC cell bank.

### 2.14 Quantitative real-time polymerase chain reaction (qRT−PCR)

mRNA was extracted from tissues with TRIzol reagent (Invitrogen, Carlsbad, CA, USA) according to the manufacturer’s protocol. The SYBR Green PCR Master One-Mix Kit (TransGen, Beijing, China) was used for qPCR to determine mRNA expression. Detailed information about all the primers used is listed in **Supplementary Table 12**.

### 2.15 Wound healing assay

Approximately 3×10^5^ A2780 and SKOV3 OC cells were seeded in a 6-well plate. After the cells filled the entire area, the culture inserts were removed. The cells were treated with PB (1.0 or 2.0 µM) or DMSO for 48 h. The cells were then rinsed twice with PBS to remove floating cells. Images were obtained under an optical microscope (ix71, Olympus, Japan) at 0 h, 24 h and 48 h after wound induction.

### 2.16 Western blot analysis

The details of the assay were as described in our previous study (16). The antibodies used are listed in **Supplementary Table 12**.

### 2.17 Immunohistochemical analysis

The details of the assay were as described in our previous study (17). The antibodies used are listed in **Supplementary Table 12**. When tissue slices are observed under an optical microscope, they are graded based on the degree of staining and the extent of positivity. The degree of staining can range from 0 to 3, representing varying depths of color (negative staining, pale yellow, light brown, and dark brown). The extent of positivity can range from 1 to 4, representing different percentages of positive cells (0-25%, 26-50%, 51-75%, 76-100%). By grading the intensity of cellular staining and the percentage of positive cells, these two scores are multiplied to obtain the final score.

### 2.18 Plasmid construction and transfection

Stably transfected small interfering RNAs (siRNAs) were obtained from ElifeBio (Hangzhou, China) and transfected into cells via Lipofectamine iMax (Invitrogen, AL, USA) following the manufacturer’s instructions. The transfection efficiency was verified via qRT−PCR.

### 2.19 Transwell assays

The premixed matrix gel was added to the Transwell chamber, which was then placed in a 24-well deep-well plate and incubated in a cell culture incubator for 2 hours. Adherent cells were digested with trypsin solution, resuspended by pipetting, and counted via a cell counter. OC cells transfected with GAS1 were digested and seeded in the upper chamber, which was supplemented with 0.2 ml of serum-free medium. Then, 700 µl of complete medium containing serum was added to the 24-well deep-well plate housing the Transwell chamber. After 24 hours, the Transwell chamber was removed, and the cells were processed for subsequent fixation, staining, and counting.

### 2.20 Cell proliferation analysis

We evaluated the proliferation of OC cells transfected with GAS1 via colony formation and EdU assays. The specific experimental procedures were performed as described previously (18).

### 2.21 Description of the statistical analysis

For statistical mapping, the Wilcoxon test was used to compare the differences between two groups of samples, and the Kruskal−Wallis test was used to compare the differences between multiple groups of samples.

## 3 Results

### 3.1 Single-cell landscape and cuproptosis activity in OC

#### 3.1.1 Expression of the TME and CRGs in OC

**Figure 1:**
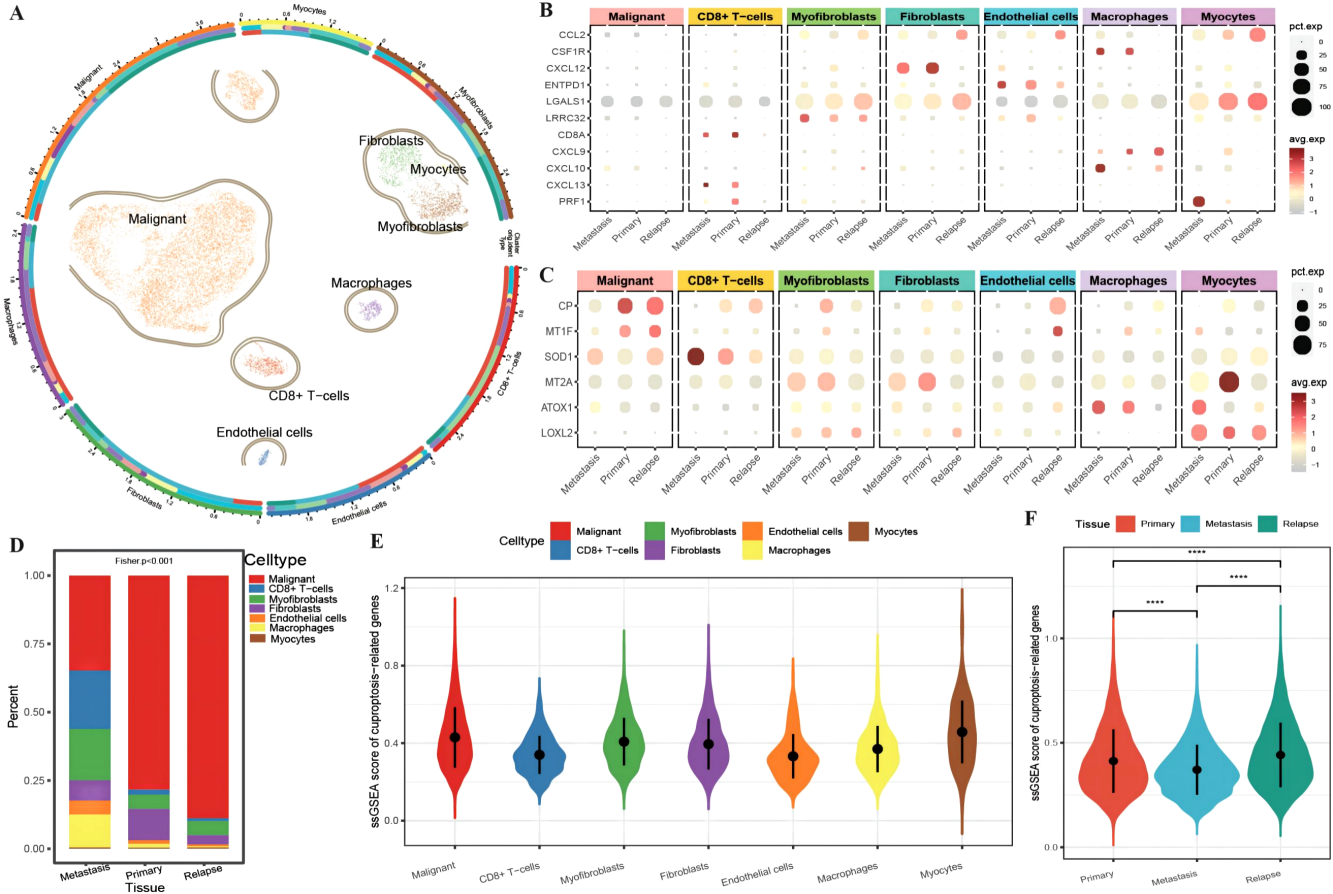
Differences in the tumor microenvironment at the single-cell level. **(A)** UMAP distribution map of cell clustering and annotation results, where the circles represent cell annotation results. From outer to inner, they respectively indicate cell type, sample source, and tissue type; **(B)** Bubble plot of the expression of anti-tumor immune and anti-tumor response genes combined with different cell types from various tissue sources, with the size of the dots representing the number of cells expressing the genes and the color indicating the level of expression; **(C)** Bubble plot of the expression of ferroptosis-related marker genes combined with different cell types from various tissue sources, with the size of the dots representing the number of cells expressing the genes and the color indicating the level of expression; **(D)** Bar chart depicting the distribution of cell types in different tissues, with different colors representing different cell types; **(E)** Violin plot showing the enrichment scores of ferroptosis-related genes in various cell clusters; **(F)** Violin plot illustrating the differences in ferroptosis enrichment scores among primary tissue, metastatic tissue, and recurrent tissue. The blue color represents cells from metastatic tissue, red indicates cells from primary cancer tissue, and green represents cells from recurrent tissue. ****p < 0.0001.

A total of 13,511 cells from OC tissue in GSE130000 (**Table 3**) were obtained. For initial data dimensionality reduction analysis (PCA), we selected the top 15 PCs for subsequent PCA and then drew a clusterree (clustree.res.pdf) to annotate the cell subtypes with SingleR (**Supplementary Table 1**: cell_info). We classified all epithelial cells, including seven cell subtypes, in tumor tissue as a malignant cell group (**Figure 1A**). To investigate the differences in the immune function of different cell types in different tissues, we first plotted an expression bubble chart of antitumor response factors and antitumor immune genes, which revealed low expression levels of these genes in malignant cells, whereas the antitumor response factor in CD8+ T cells was active in both metastatic and primary cancer tissues. Moreover, antitumor immune factors from macrophages, fibroblasts, and endothelial cells exhibited much greater activity in primary and metastatic tissues than in recurrent tissue (**Figure 1B**). We also plotted an expression bubble chart of the CRGs, which revealed that CP was expressed at higher levels in the malignant cells of primary and recurrent tissues and that MT2A was expressed at higher levels in the primary myocardial cells. However, SOD1 preferred CD8+ T cells in metastatic tissue (**Figure 1C**). Next, we explored the distribution of different cell types across different tissue sources, revealing the diverse proportions of malignant cells, CD8+ T cells, myofibroblasts and macrophages in different tissues (**Figure 1D**). Finally, according to the expression of each gene in single cells, we calculated the ssGSEA scores of the CRGs (**Supplementary Table 1**: cuproptosis_ssGSEA), which revealed that the enrichment level of CRGs in malignant cells was relatively high (**Figure 1E**). Moreover, the enrichment score of CRGs in recurrent tissue was greater than that in primary and metastatic tissue (**Figure 1F**).

#### 3.1.2 CRGs associated with cellular immunity in OC

**Figure 2:**
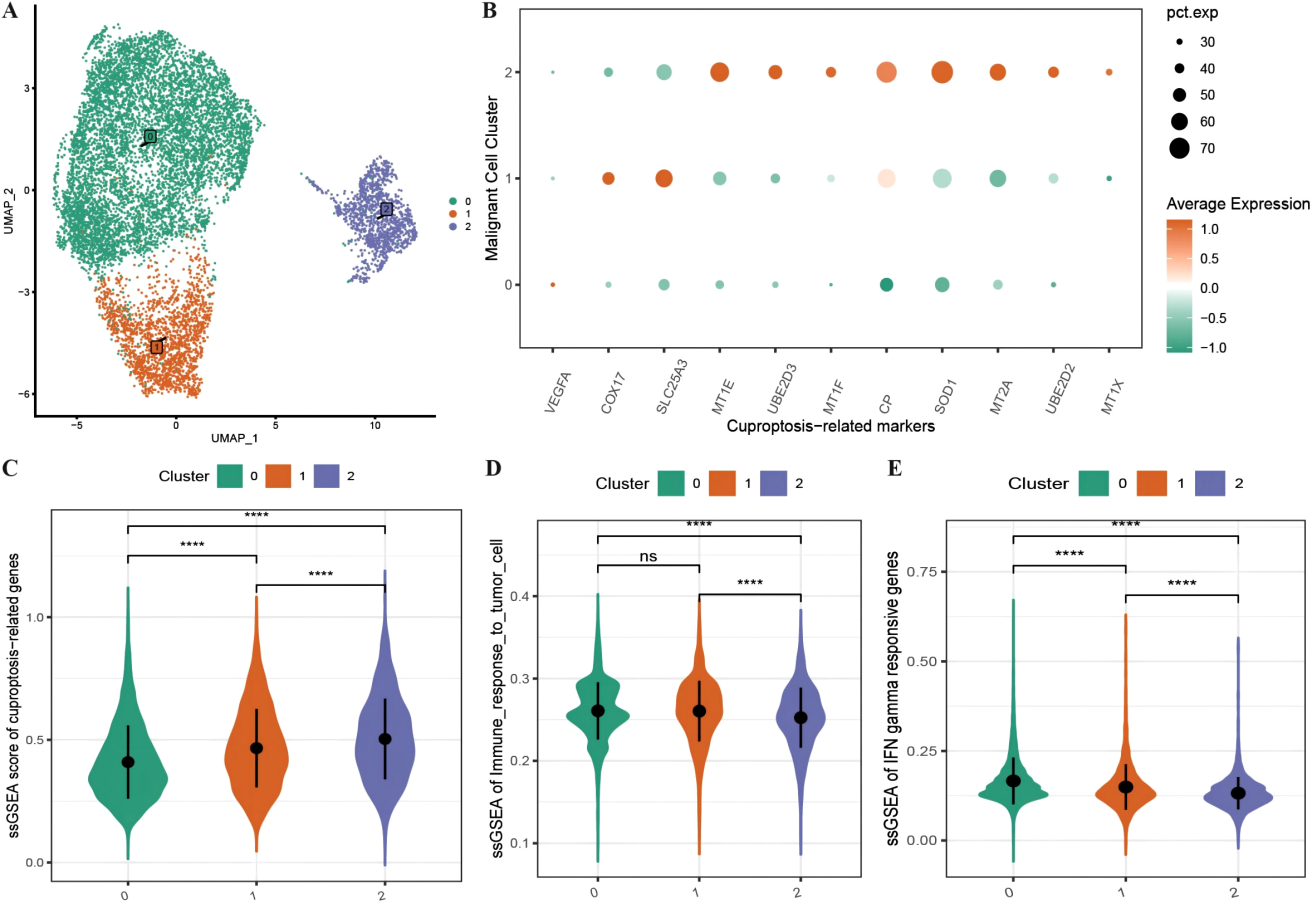
Characteristics of cuproptosis in malignant cells. **(A)** UMAP distribution map of the re-clustering results of malignant cells; **(B)** Bubble plot showing the expression of all ferroptosis-related marker genes in malignant subtypes, where the size of the dots represents the number of cells expressing the genes and the color represents the level of expression; **(C)** Violin plot displaying the differences in enrichment scores of ferroptosis-related gene sets among malignant subtypes; **(D, E)** Violin plots illustrating the differences in enrichment scores of tumor cell immune response and interferon response gene sets among malignant subtypes. ****p < 0.0001.

We subdivided the 10,358 malignant cells into three malignant subgroups (**Figure 2A**) and then generated an expression bubble map of all the CRGs by identifying the marker genes of each cluster, which revealed that the expression of CRGs was significantly greater in subgroup 2 than in subgroups 1 and 0 (**Figure 2B**). Moreover, the enrichment scores of the CRGs were highest in subgroup 2, followed by subgroups 1 and 0 (**Figure 2C**). We also performed immune functional analysis of the three malignant subtypes, which revealed that the enrichment scores for tumor cell immune response ability and interferon-γ response genes were significantly greater in subgroup 2 than in subgroups 1 and 0 (**Figures 2D, E**).

#### 3.1.3 Identification of cuproptosis activity in malignant cells

**Figure 3:**
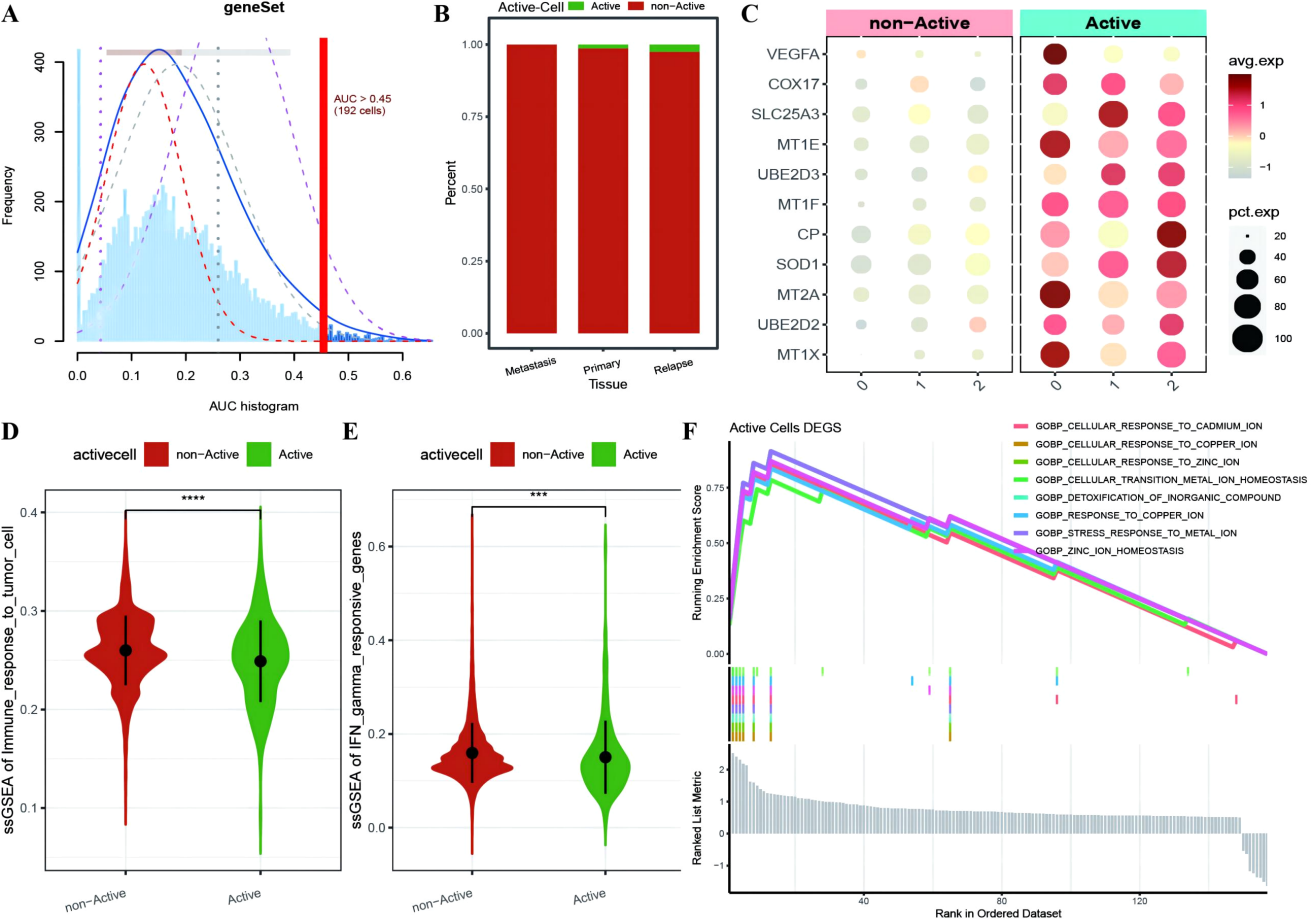
Identification of active cuproptosis malignant cell population. **(A)** Single-cell AUC scores for ferroptosis genes, with the optimal threshold being 0.45; **(B)** Bar chart displaying the distribution of active populations in different tissue types, where red represents inactive populations and green represents active populations; **(C)** Bubble plot depicting the expression of ferroptosis marker genes in various malignant cell subtypes and different activity types, with a redder color indicating higher average expression values and the size of the dots representing the number of expressing cells; **(D, E)** Violin plots showing differences in immune function between active and non-active malignant cell populations with respect to ferroptosis activity, where green represents the active population and red represents the non-active population; **(F)** Line plot illustrating the enrichment results of differentially expressed genes between the active and non-active ferroptosis groups based on GOBP-GSEA. ***p < 0.001, ****p < 0.0001.

To further investigate the expression and functional characteristics of CRGs at the single-cell level, we identified 192 cells as an active malignant population related to active cuproptosis in malignancies (**Figure 3A**) and used the optimal threshold to determine cell viability (**Supplementary Table 2**: cell_info). A cumulative distribution histogram was subsequently plotted to display the distribution of active and inactive cuproptotic malignant cells in each tissue type, which revealed that the number of active cuproptotic malignant cells was highest in the recurrent tissue (**Figure 3B**). In addition, a bubble chart of CRGs in three subtypes and different active types revealed that CRGs were enriched in the active population of all malignant subtypes, especially in subtype 2 (**Figure 3C**). To identify functional differences between the active and inactive populations, we compared their immune functions and identified the active population with a significantly lower level of immune response to tumor cells and interferon-γ response gene enrichment scores compared with the inactive population (**Figures 3D, E**). Finally, differential expression analysis was conducted on the two cell populations, with 157 DEGs identified (**Supplementary Table 2**: Active_cells_DEGS), which were enriched mainly in response to metal ions such as copper, zinc, and cadmium, as well as in the detoxification of organic compounds, according to GOBP enrichment analysis (**Figure 3F**).

### 3.2 Role of the identification of molecular subtypes associated with active necroptosis DEGs

#### 3.2.1 Immune infiltration between molecular subtypes in OC

**Figure 4:**
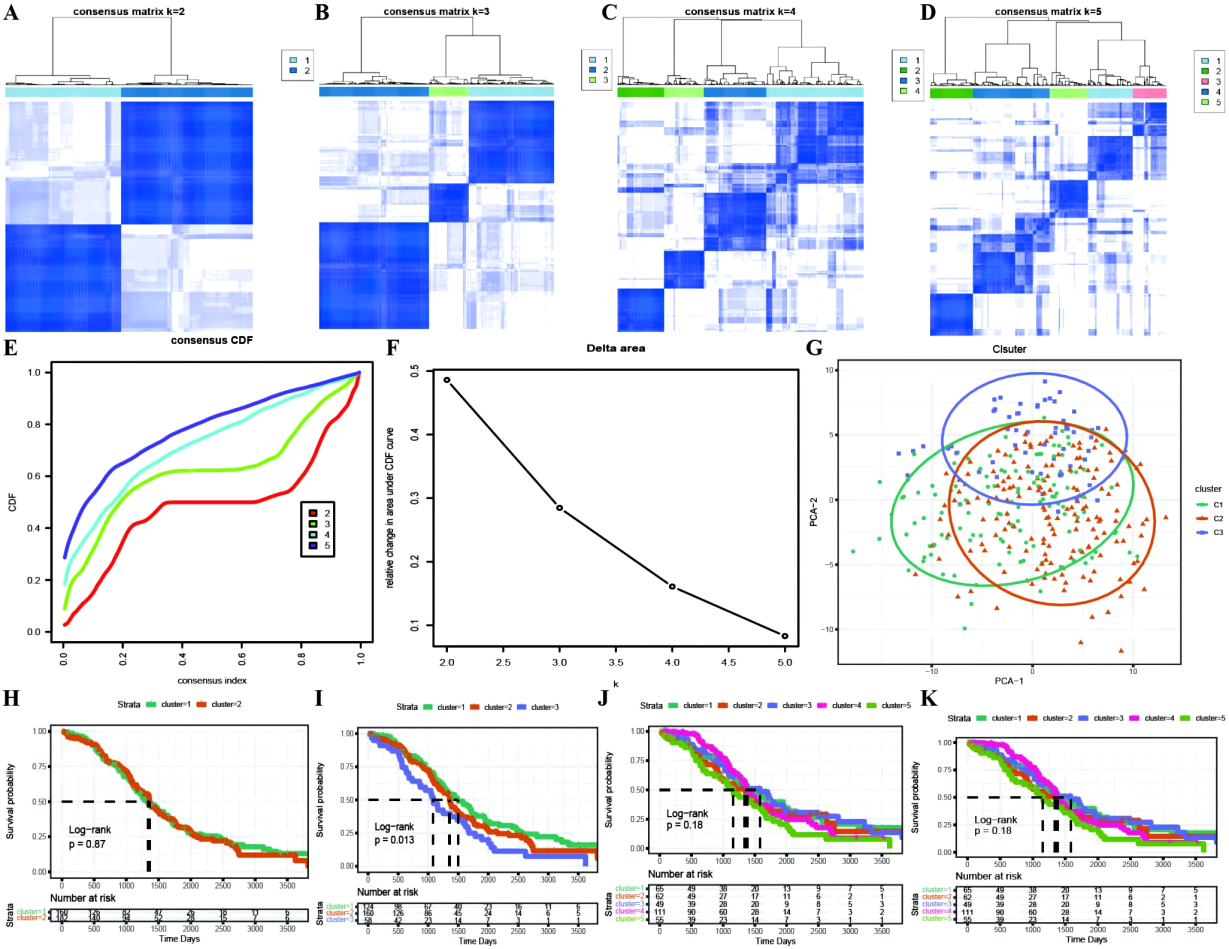
Identification of TCGA molecular subtypes in OC. **(A–D)** Clustering results for different numbers of clusters, specifically k=2, k=3, k=4, and k=5; **(E)** Distribution of the cumulative distribution function (CDF) curve for consensus clustering; **(F)** Distribution of the area under the cumulative distribution function (CDF) curve for consensus clustering; **(G)** Scatter plot showing the results of clustering using PCA dimensionality reduction algorithm, where green represents C1, orange represents C2, and purple represents C3; **(H–K)** Survival curves for different numbers of clusters (k=2, k=3, k=4, k=5), where different colored curves represent different clusters.

Based on the identification of DEGs between active and inactive cuproptotic malignant cell populations via single-cell analysis, we identified OC molecular subtypes with the best clustering performance achieved via the PAM clustering algorithm, Spearman distance, and K=3 (**Figures 4A–D**; **Supplementary Table 3**). The consistency clustering cumulative distribution function (CDF) with the KM curves revealed clear boundaries between the three subtypes, indicating good clustering results, and K=3 was the result of our molecular subtype identification (**Figures 4E–K**; **Supplementary Table 3**: cc_group). We also explored the different cuproptosis expression patterns of the three subtypes (**Supplementary Figure 1**).

**Figure 5:**
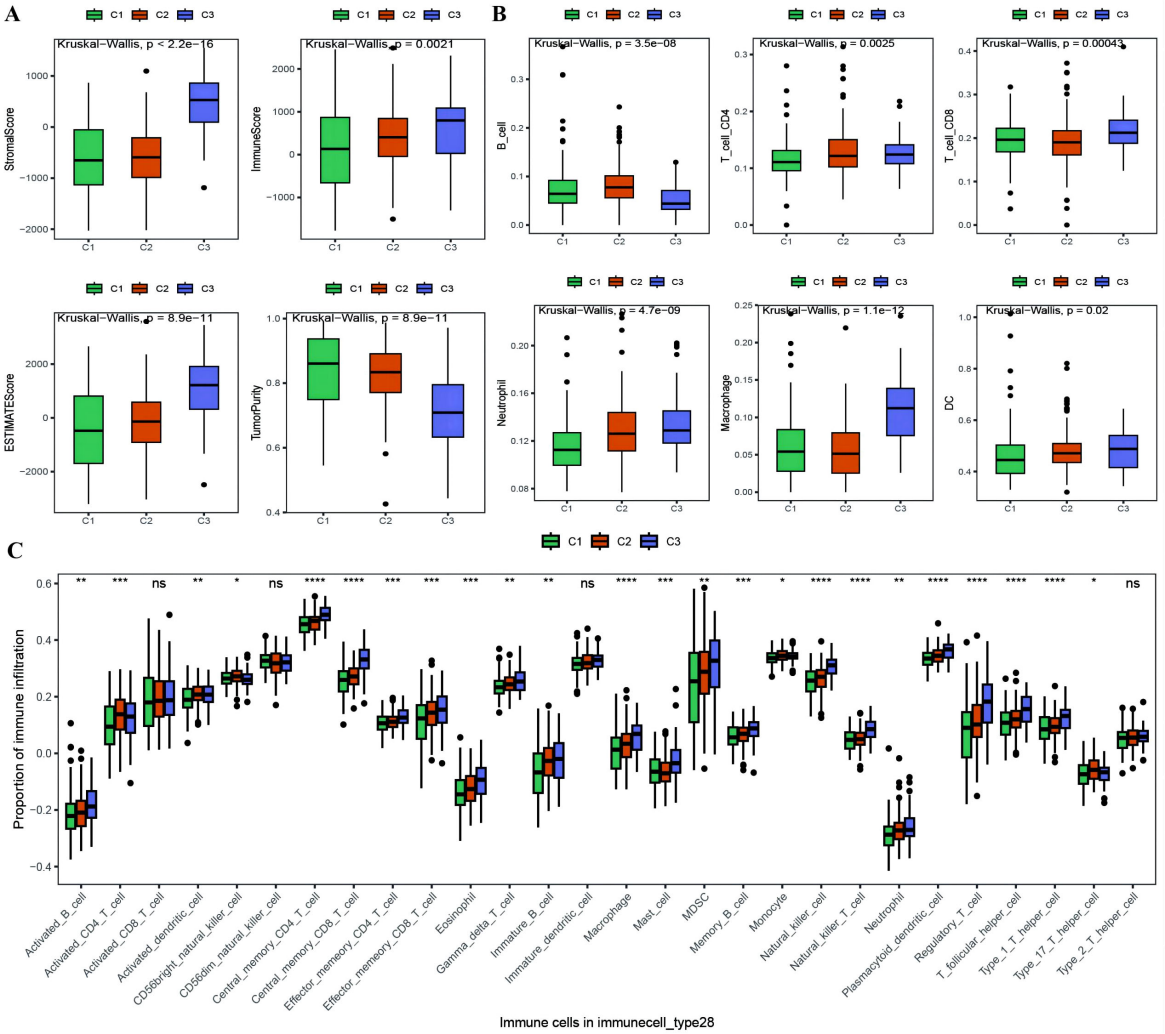
Differences in proportion of immune-infiltrating cells among TCGA molecular subtypes. **(A)** Box plot showing the differences in ESTIMATE scores between molecular subtypes of ovarian cancer, where green represents C1, orange represents C2, and purple represents C3; **(B)** Box plot illustrating the differences in the proportions of immune infiltrating cells between molecular subtypes calculated using the TIMER algorithm; **(C)** Box plot displaying the differences in the proportions of 28 types of immune infiltrating cells between molecular subtypes. *p < 0.05, **p < 0.01, ***p < 0.001, ****p < 0.0001.

**Figure 6:**
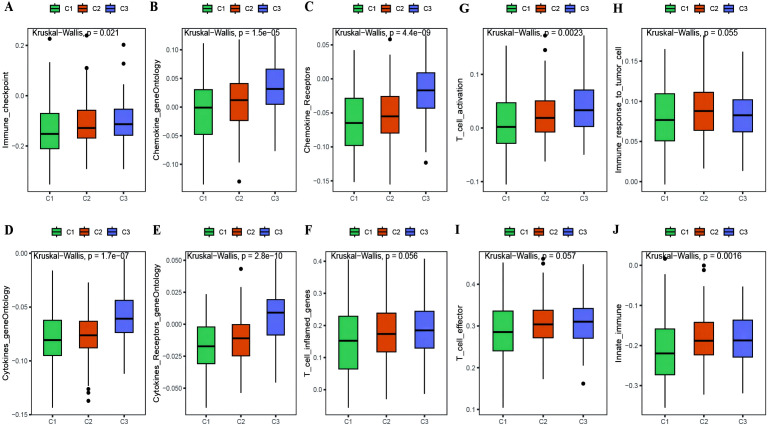
Differences in immune enrichment among subtypes. **(A–F)** Box plots showing the differences in enrichment of immune factor gene sets between subtypes, including immune checkpoints, chemokine gene ontology, chemokine receptor gene ontology, cytokine gene ontology, cytokine receptor gene ontology, and T cell inflammatory genes; **(G–J)** Box plots illustrating the differences in immune functions between subtypes, including T cell activation, immune response to tumor cells, effector T cells, and innate immunity.

With the results of the immunoreactive cell proportion analysis of the TCGA OC samples (**Supplementary Table 4**), we analyzed the proportion of immunoreactive cells among the three subtypes, which revealed that the stromal, immune, and ESTIMATE scores of the C3 subtype were significantly greater than those of the other two subtypes, whereas the tumor purity was lower (**Figure 5A**). Moreover, the B-cell infiltration level was lower in the C3 subtype than in the other subtypes, whereas the infiltration of T cells, macrophages, DCs, and neutrophils was greater according to the TIMER algorithm (**Figure 5B**). Finally, we explored the infiltration proportions of 28 immune cells among the three subtypes via the ssGSEA algorithm and found that there were significant differences in the infiltration proportions of 24 cell types and that the infiltration proportion of the C3 subtype was significantly greater than those of the other two subtypes (**Figure 5C**). Furthermore, we also detected differences in the enrichment scores of the six immune-related gene sets among the three MSs, from which we found that the immune factor enrichment score in C3 was higher than that in C1 and C2 and that C2 had a higher score than C1 (**Figures 6A–F**). Additionally, the enrichment of four immune function gene sets among the subtypes revealed that T-cell activation and innate immunity in C3 were significantly stronger than those in C2 and C1 (**Figures 6G–J**).

#### 3.2.2 Differential expression of immune markers across molecular subtypes

The expression levels of immune checkpoints from the TISIDB database in the C3 subtype were generally significantly greater than those in the C1 and C2 subtypes (**Supplementary Figure 2A**). Second, to explore the differences in other biological functions between molecular subtypes, we identified the upregulated genes in each subtype relative to those in the other two subtypes and then combined the three upregulated gene sets as the DEGs between subtypes. A total of 1033 subtype-specific DEGs were obtained (**Supplementary Table 3**: All_diff_gene). The KEGG pathway results revealed that the subtype-specific DEGs were enriched mainly in ECM−receptor interactions, proteoglycans in cancer, the PI3K−Akt signaling pathway, protein digestion and absorption, complement and coagulation cascades, phagosomes, and *Staphylococcus aureus* infection (**Supplementary Figure 2B**). Moreover, the GO functional enrichment results revealed that the DEGs were enriched mainly in biological processes related to cell tissue, migration, adhesion, regulation of vasculature development regulation, and wound healing; molecular functions such as extracellular matrix structural constituents, glycosaminoglycan binding, and growth factor binding; and cellular component gene sets such as the collagen-containing extracellular matrix, endoplasmic reticulum lumen, and collagen triple helix complex (**Supplementary Figures 2C–E**).

### 3.3 Construction and validation of a prognostic risk model for OC

**Figure 7:**
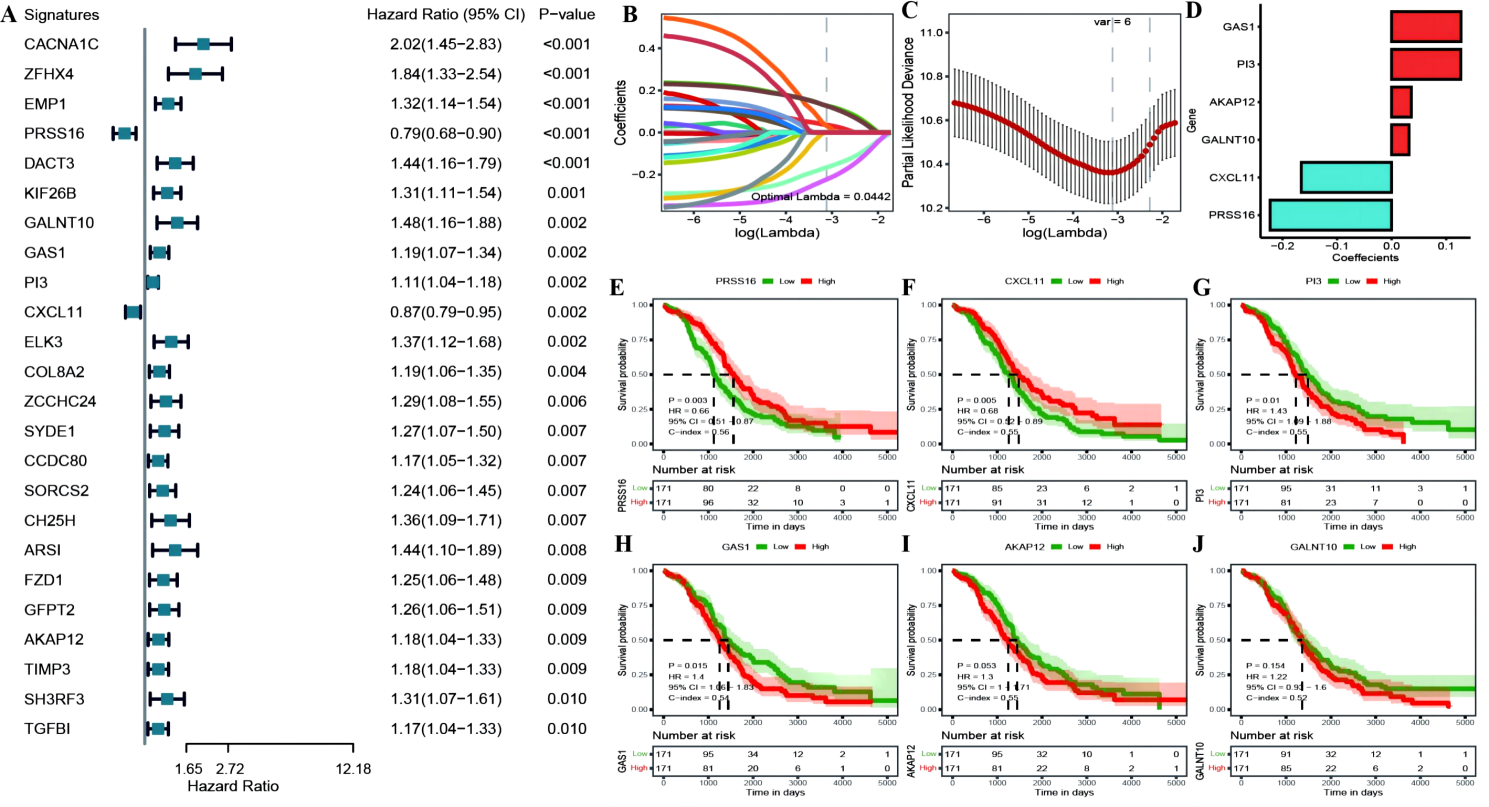
**(A)** construction of the prognostic risk model. **(A)** Forest plot of the results of single-factor Cox analysis of differentially expressed genes between subtypes; **(B)** Trajectory of changes in the independent variables of LASSO regression, where the x-axis represents the logarithm of the independent variable Lambda and the y-axis represents the coefficient of the independent variable; **(C)** Confidence interval for each Lambda in LASSO regression; **(D)** LASSO regression coefficients for 8 key prognostic factors; **(E–J)** KM curves for model genes, where red represents high expression group and green represents low expression group.

We identified 24 genes significantly associated with OC prognosis from the DEGs associated with OC among the cuproptosis subtypes (**Figure 7A**; **Supplementary Table 5**: cox_res). We subsequently applied Lasso linear regression to eliminate redundant genes according to these 24 genes, resulting in six prognosis-related signatures (**Figures 7B–D**; **Supplementary Table 5**: lasso_res). Then, we established Kaplan−Meier survival curves for these genes in the overall TCGA cohort. We found that there were significant differences between the KM curves for four genes: PRSS16 and CXCL11 had better prognostic values in the high-expression group, whereas PI3 and GAS1 had better prognostic values in the low-expression group (**Figures 7E–J**). Furthermore, we calculated the RS for each sample via the formula RiskScore = PRSS16*-0.223 + CXCL11*-0.166 + PI3*0.127 + GALNT10*0.032 + GAS1*0.127 + AKAP12*0.036, which led to high-risk and low-risk groups via the median risk score of 0.4525444 as the threshold (**Supplementary Table 5**: TCGA_Train). These six model genes were diverse in the high- and low-risk groups of the training set and were related to prognosis (**Supplementary Figures 3A–D**). The receiver operating characteristic (ROC) curve of the prognostic signature, with area under the ROC curve (AUC) values of 0.709, 0.711, and 0.773 at 3, 5, and 8 years, revealed good predictive performance of the model score (**Supplementary Figure 3E**). Similar results were also validated in the TCGA test set, TCGA_OC dataset, and GEO dataset GSE26712 (**Supplementary Figures 4**–**6**).

### 3.4 Association between RS and multiple features in OC

#### 3.4.1 Association between risk score and immune microenvironment in OC

**Figure 8:**
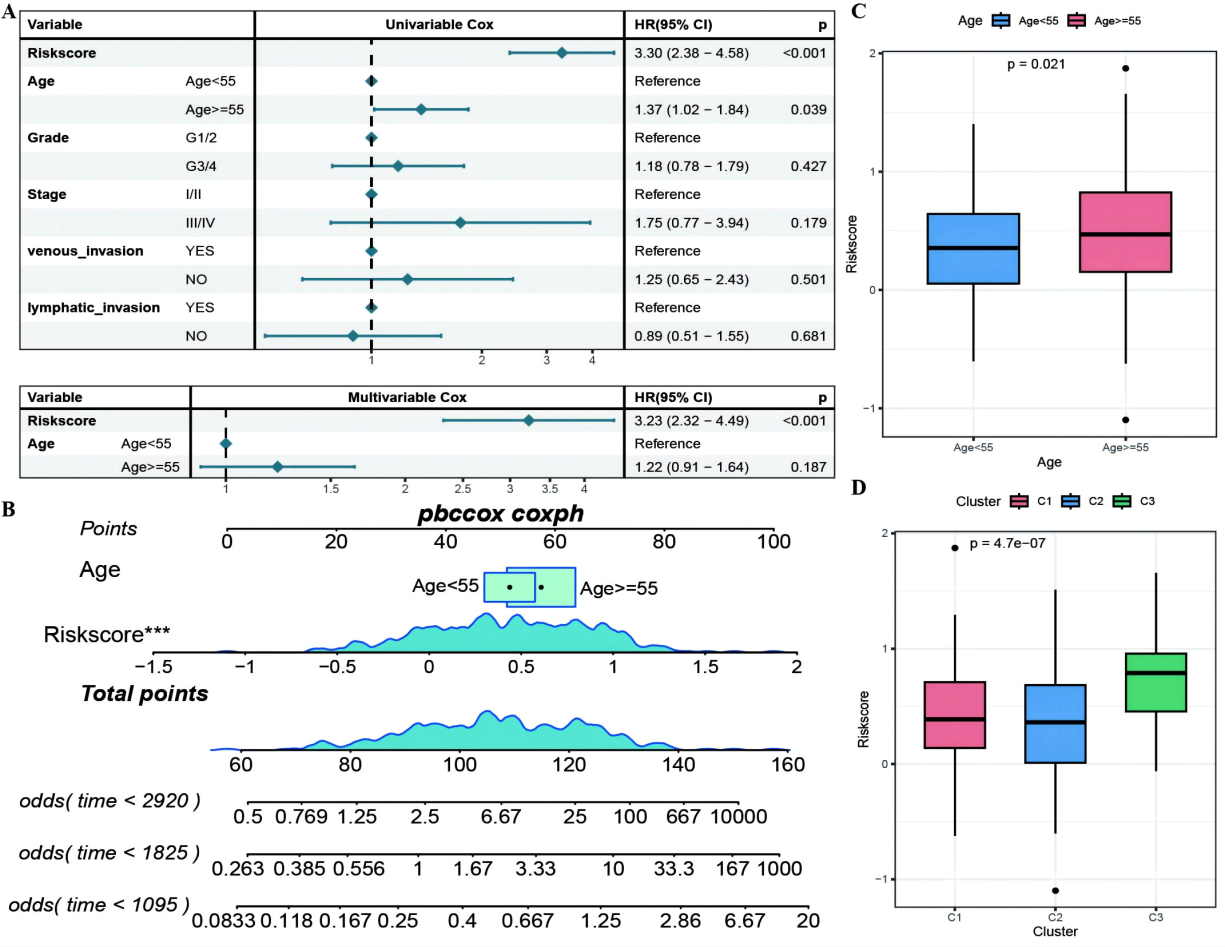
The RiskScore as an independent prognostic factor. **(A)** Forest plot of the results of single- and multi-factor Cox analysis for clinical factors; **(B)** Nomogram of the predictive model, where the square plus line segment represents the contribution of each clinical factor to the outcome event, Total Points represents the total score obtained by adding up the scores for all variables, and the three lines at the bottom represent the 8/5/3-year survival probabilities corresponding to each value point; **(C, D)** Box plots showing the differences in risk scores distribution between different Age and molecular subtype groups, with different colors representing different groups, and the p-value indicating the significance of the difference. ***p < 0.001.

**Figure 9:**
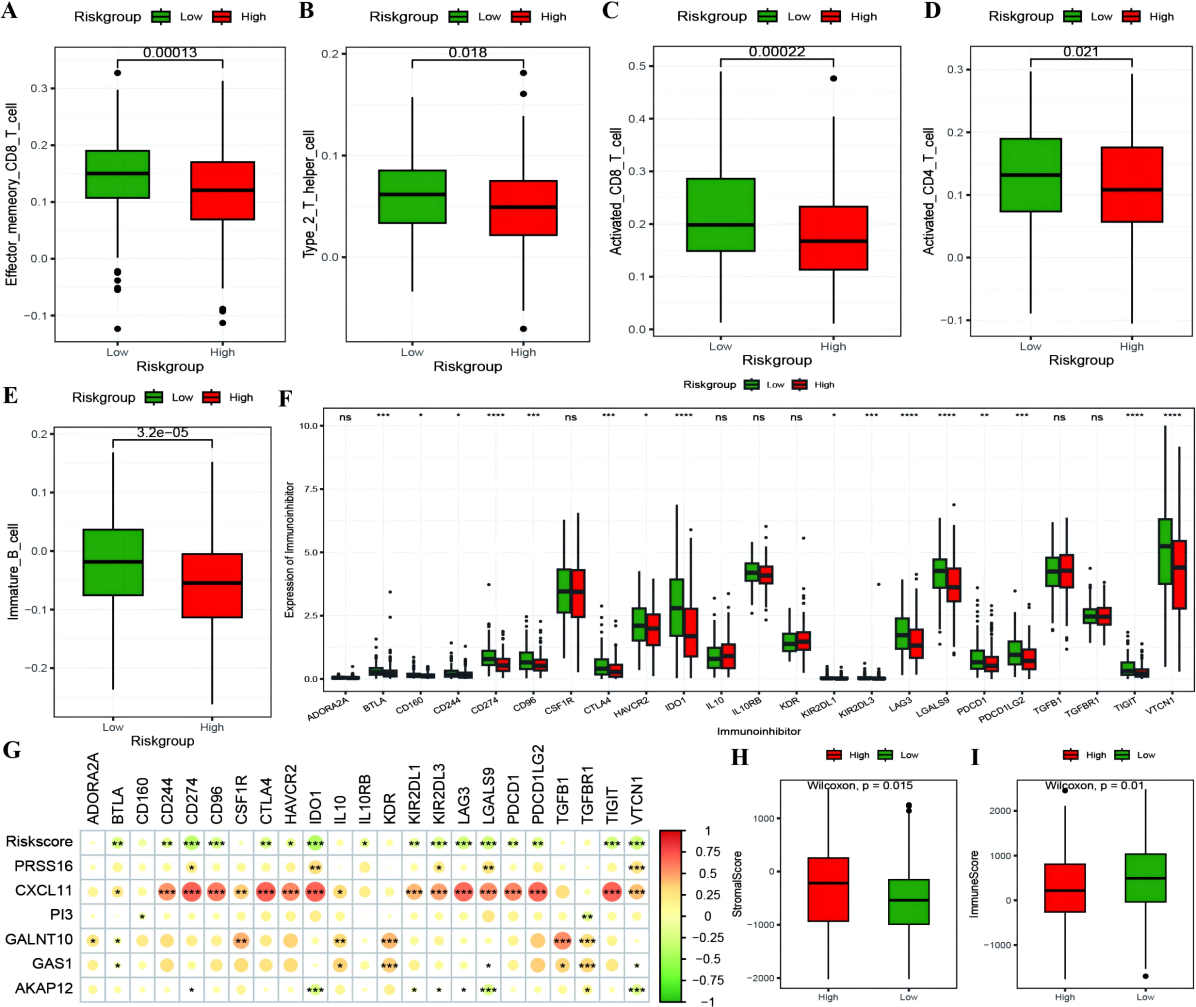
The correlation between RiskScore and immune checkpoints. **(A–E)** Box plots showing the differences in proportions of immune-infiltrating cells between high and low-risk groups calculated using the ssGSEA algorithm, where red represents the high-risk group and green represents the low-risk group; **(F)** Box plot illustrating the expression differences of 23 immune checkpoint inhibitors between high and low-risk groups, where red represents the high-risk group and green represents the low-risk group; **(G)** Heatmap of the correlation coefficients between the expression of model genes, risk scores, and the expression of immune checkpoint inhibitors, where the color of the dots represents the strength of the correlation, and “*” denotes significance; **(H, I)** Box plots displaying the differences in stromal score and immune score between high and low-risk groups. *p < 0.05, **p < 0.01, ***p < 0.001, ****p < 0.0001.

The RS was confirmed to be an independent prognostic factor for age (>55), stage and venous invasion in OC patients (**Figure 8A**; **Supplementary Table 5**: Clinical_stat). The RS is a clinical factor that contributes to survival time and survival status in combination with the clinical indicator age (**Figure 8B**). The risk score was significantly different between both the age subgroups and the molecular subtypes (**Figures 8C, D**), indicating that the age and molecular subtypes of the ovarian cancer samples were significantly associated with the RS. To investigate the differences in the tumor immune microenvironment between the high- and low-risk groups in the RS, in light of the estimation results of the proportion of infiltrating immune cells, we discovered that 5 of the 28 immune cells, including activated CD4+ T cells, activated CD8+ T cells, effector memory CD8+ T cells, immature B cells, and type 2 T helper cells, were significantly greater in the low-RS group than in the high-RS group according to the ssGSEA algorithm (**Figures 9A–E**). We subsequently explored the expression of 23 immunosuppressive checkpoints (**Supplementary Table 6**: check_model_data) in the high- and low-risk groups, 16 of which were significantly more highly expressed in the low-risk group than in the high-risk group (**Figure 9F**). In addition, the associations between the expression of 6 genes from the RS and that of 23 immunosuppression checkpoint genes (**Supplementary Table 6**: corrdata & corrp) revealed that the expression of CXCL11 was significantly positively correlated with that of multiple immune checkpoint genes; however, the expression of the RS was generally negatively correlated with that of other genes (**Figure 9G**). Finally, the stromal score (**Figure 9H**) from the ESTIMATE algorithm was higher in the high-RS group than in the low-RS group, and the immune score (**Figure 9I**) results were the opposite.

Analysis of the differences in the expression of antitumor immune and antitumor response genes in the RS groups revealed that the expression of antitumor response-related genes was significantly lower in the high-risk group than in the low-risk group. We then selected 197 DEGs between the two groups (**Supplementary Table 7**) to explore their functions via Kyoto Encyclopedia of Genes and Genomes (KEGG) and Gene Ontology (GO) enrichment analyses, which revealed that the DEGs were enriched mainly in antigen processing and presentation, Epstein–Barr virus (EBV) infection, allograft rejection, autoimmune thyroid disease and other Kyoto Encyclopedia of Genes and Genomes (KEGG) pathways, as well as in gene ontology (GO) functional gene sets related to antigen processing, processing and presentation, response to viruses, and major histocompatibility complex (MHC) protein complexes.

#### 3.4.2 Correlation of the RS model with the HALLMARK

Based on the HALLMARK enrichment score results of the OC samples and the RS information, we aimed to explore the pathway enrichment differences between the high- and low-risk groups (**Supplementary Table 8**), and 28 of the pathways presented significant differences in enrichment scores between the high- and low-risk groups (**Supplementary Figure 6A**). Moreover, the expression of CXCL11 and GALNT10 was significantly positively correlated with multiple pathway enrichment scores; however, the expression of RS was significantly negatively correlated with the interferon α and γ response (**Supplementary Figure 6B**). RS was found to be significantly negatively correlated with multiple immune function gene sets, where we selected four functional gene sets with strong correlations, including Co inhibition, interferon γ response, immune response to tumor cells and antigen processing and presentation (**Supplementary Figures 6C–F**).

#### 3.4.3 Association between RS and tumor genome mutations

**Figure 10:**
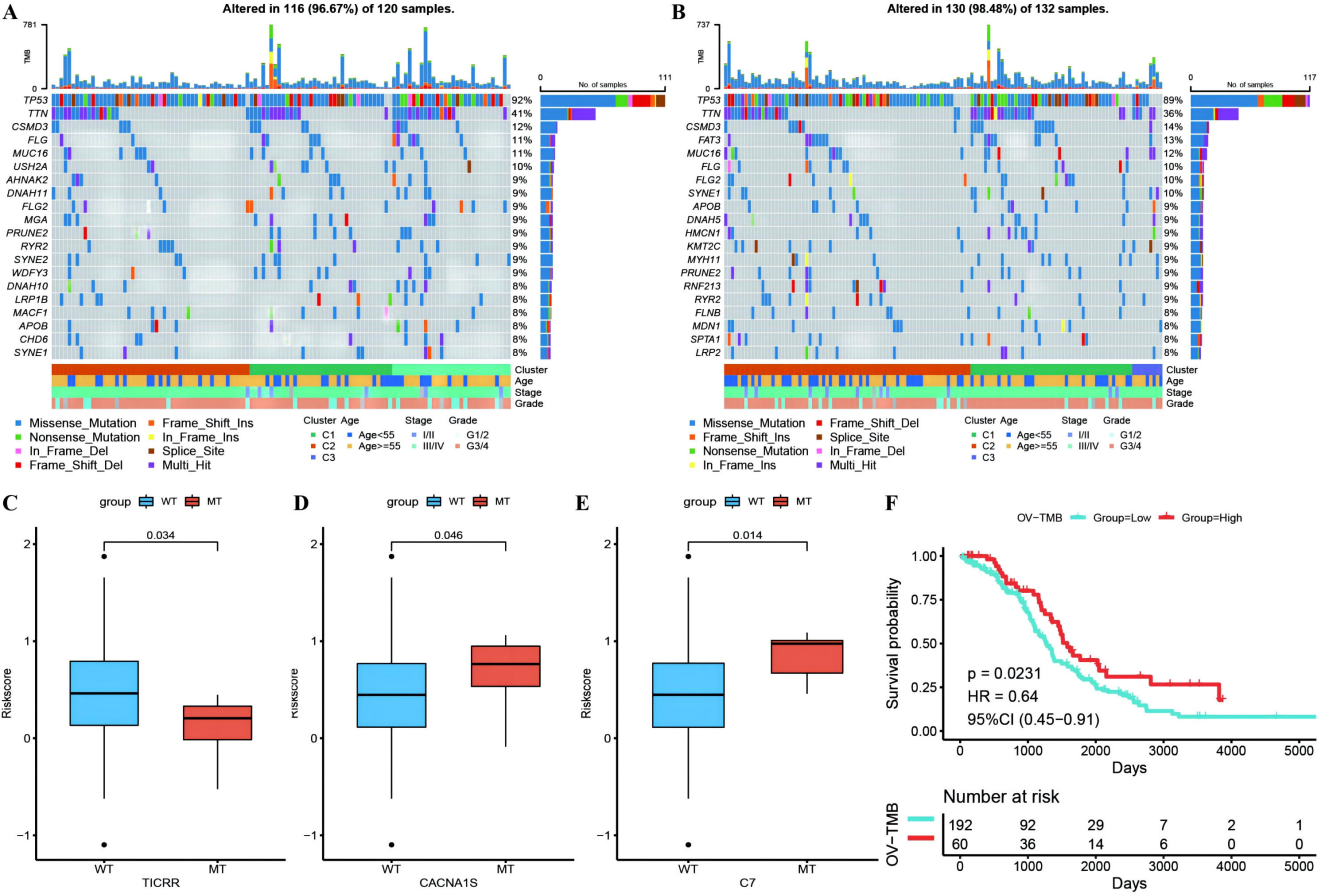
Differences of gene mutant enrichment in Riskscore model. **(A)** Waterfall plot of the top 20 genes’ SNV mutation frequencies in the high-risk group; **(B)** Waterfall plot of the top 20 genes’ SNV mutation frequencies in the low-risk group; **(C–E)** Box plots illustrating the difference in Riskscore between mutated and wild-type groups for genes with significantly different mutation frequencies between high and low-risk groups, where red represents the mutated group and blue represents the wild-type group; **(F)** Survival curve comparison between high and low TMB groups, where red represents the high TMB group and blue represents the low TMB group.

Waterfall plots of the top 20 genes with mutation frequencies were generated separately for the high- and low-risk groups combined with other clinical information, demonstrating the distribution of gene mutations between the two groups and samples with different clinical characteristics, with TP53, TTN, and CSMD3 having the highest mutation frequencies in OC (**Figures 10A, B**). Due to differences in gene mutation frequencies between the high- and low-risk groups (**Supplementary Table 9**: mafCompare_High_VS_Low), we divided the samples into MT and WT groups to analyze the difference in RS between them. We found that the risk scores of the TICRR, CACNA1S and C7 gene groups were significantly different from those of the wild-type group, and the mutation frequency of these genes was greater (**Figures 10C–E**). Using the same methodology, we also scrutinized the correlation between tumor mutational burden (TMB) (**Supplementary Table 9**: Riskscore_tmb_res) or RS and prognosis, revealing a more favorable prognosis associated with high TMB (**Figure 10F**).

### 3.5 RS model estimation for predicting patient clinical efficacy

**Figure 11:**
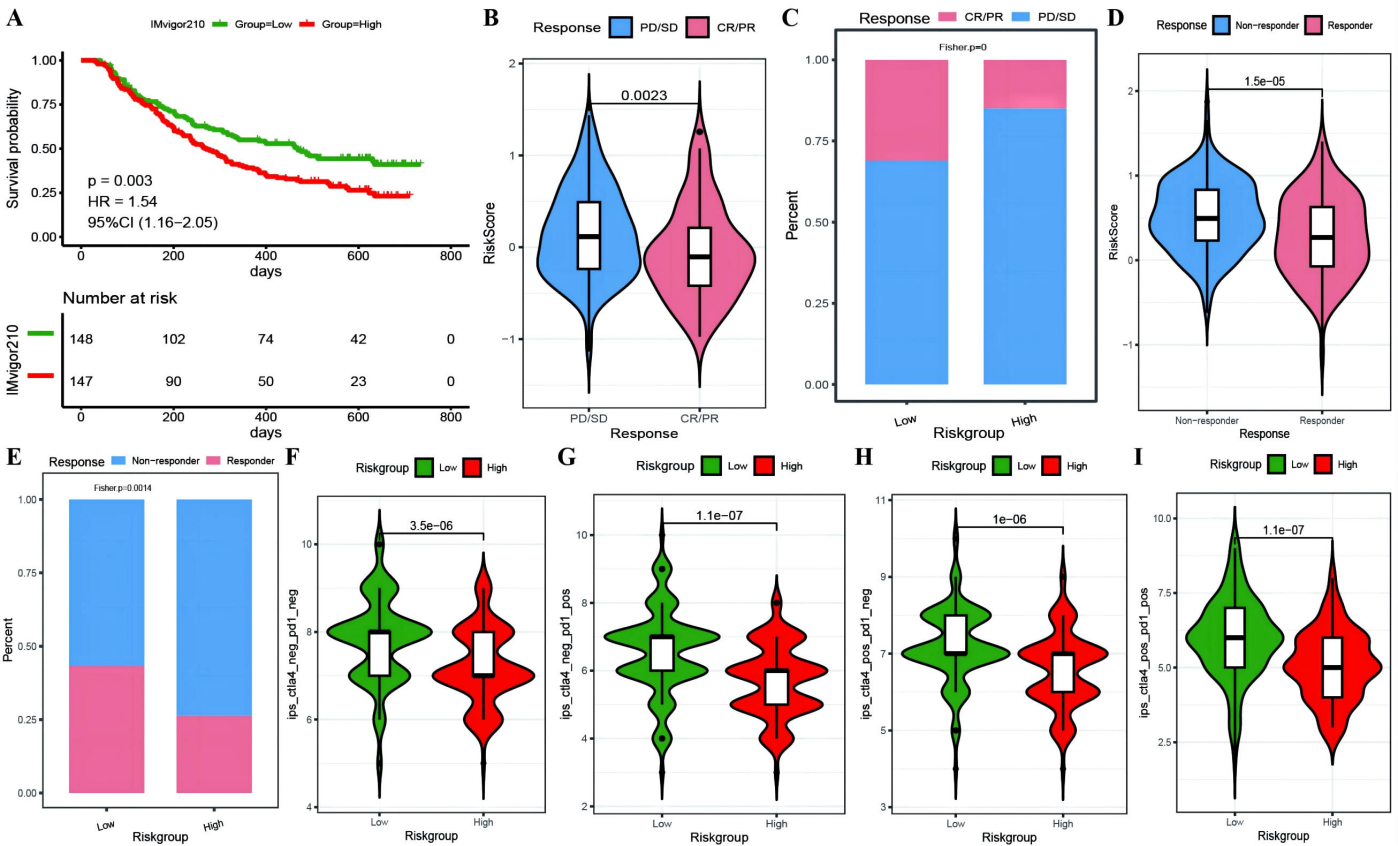
Predictive modeling of immunotherapy efficacy via risk stratification. **(A)** Kaplan-Meier curves for high-risk versus low-risk groups within the IMvigor210 cohort; **(B)** Violin plots illustrating differences in risk scores between responder and non-responder groups within the IMvigor210 cohort, with responders depicted in red and non-responders in blue; **(C)** Bar graphs showing the cumulative distribution differences between responder and non-responder groups among high-risk and low-risk categories within the IMvigor210 cohort; **(D)** Violin plots of risk scores for responder versus non-responder groups as predicted by TIDE; **(E)** Bar graphs depicting the cumulative distribution of responder versus non-responder groups among model-based stratifications as forecasted by TIDE; **(F–I)** Violin plots representing the differences in IPS scores between high-risk and low-risk groups, with high-risk groups colored in red and low-risk groups in green.

**Figure 12:**
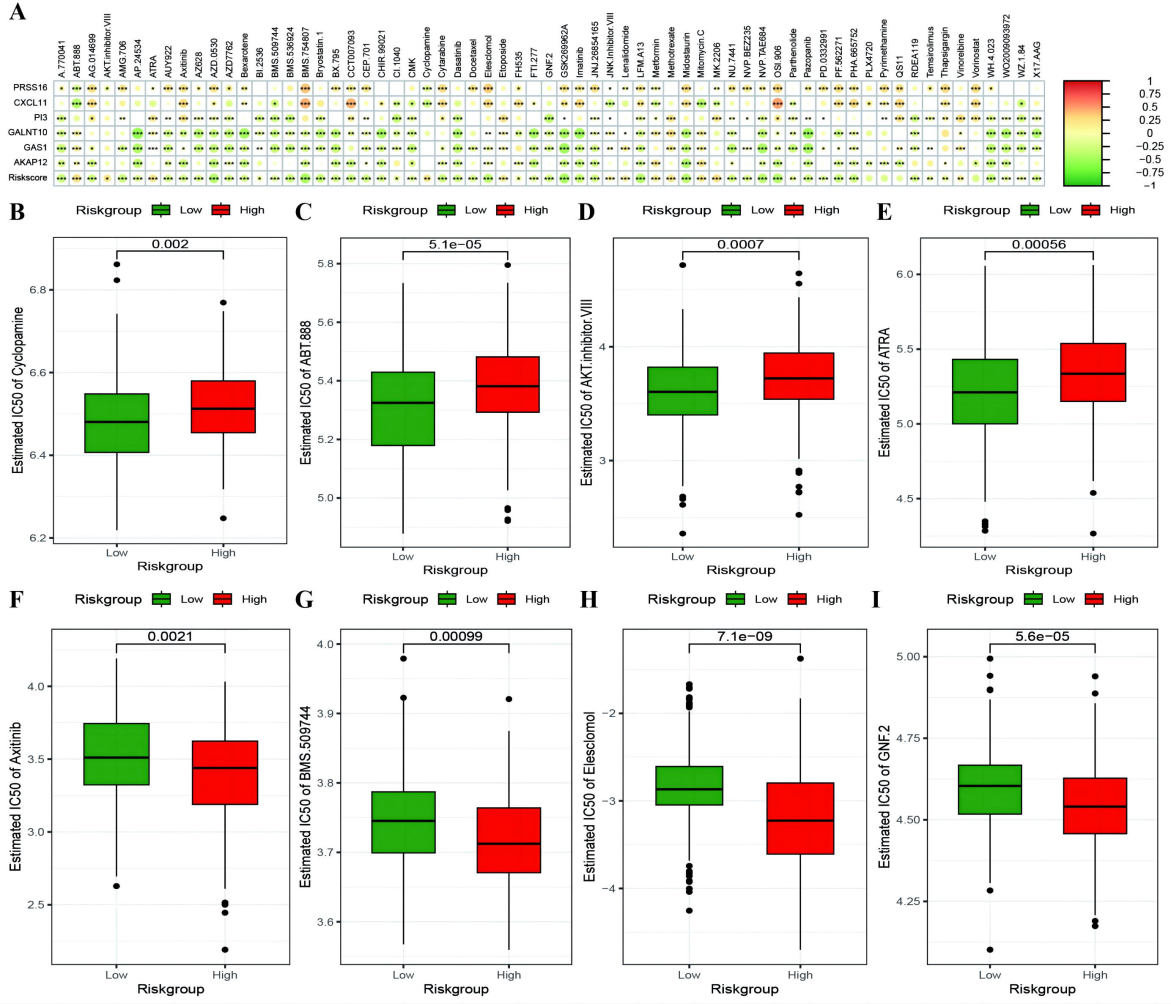
Risk model predicts chemotherapy drug resistance. **(A)** Heatmap illustrating the correlation between IC50 values of drugs showing significant differences in sensitivity between high-risk and low-risk groups, model gene expression, and RiskScore. The intensity of the color indicates the level of correlation, with * denoting significance; **(B–E)** Box plots depicting differences in IC50 values for drugs to which high-risk groups are more sensitive, with high-risk groups in red and low-risk groups in green; **(F–I)** Box plots showing differences in IC50 values for drugs to which low-risk groups are more sensitive. *p < 0.05, **p < 0.001, ***p < 0.001.

To investigate whether genes from the Riskscore model can serve as markers for immunotherapy response, we explored the ability of tumor risk scores to predict patient benefit from immunotherapy. Initially, within the immunotherapy dataset, risk scores were computed for each sample and then categorized into high- and low-risk groups (**Supplementary Table 10**: IMvigor210_res). The low-risk group had a better prognosis (**Figure 11A**), with more pronounced benefits following immunotherapy (**Figures 11B, C**). TIDE (**Supplementary Table 10**: TIDE_res) was subsequently used to predict the immune response status of samples in the TCGA_OC dataset to evaluate the model’s ability to predict the immune response. The risk score of the responsive group was significantly lower than that of the nonresponsive group (**Figures 11D, E**). Additionally, the proportion of responders in the low-RS group was significantly greater than that in the high-RS group. The analysis from TIDE indicated a significant correlation between risk scores, RS grouping, and immune response, suggesting a strong predictive ability of the model for the immune response. Furthermore, we employed the immunophenoscore (IPS) (**Supplementary Table 10**: IPS_res) to explore clinical efficacy from the perspective of tumor immunogenicity and found that all four IPS scores in the low-RS group were significantly higher than those in the high-RS group, further revealing that patients in the low-RS group are more likely to benefit from immunotherapy (**Figures 11F–I**). Furthermore, we analyzed the relationships between the risk score model and the IC50 values of targeted and chemotherapeutic drugs via the GDS and TCGA databases (**Supplementary Table 11**: drug_res&sign_stat; **Supplementary Table 11**: corr_IC50_ModelGene_corr). Our findings confirmed a significant negative correlation between the expression of GALNT10, GAS1, and AKAP12 and the risk score and IC50 values of the drugs (**Figure 12A**). Additionally, we observed a strong positive correlation between the expression of the genes CXCL11 and PRSS16 and the IC50 values of the drugs. The different treatment responses to various targeted drugs’ IC50 values were also evident between the high- and low-RS groups (**Figures 12B–I**).

### 3.6 Validation of the expression levels of hub genes in samples

**Figure 13:**
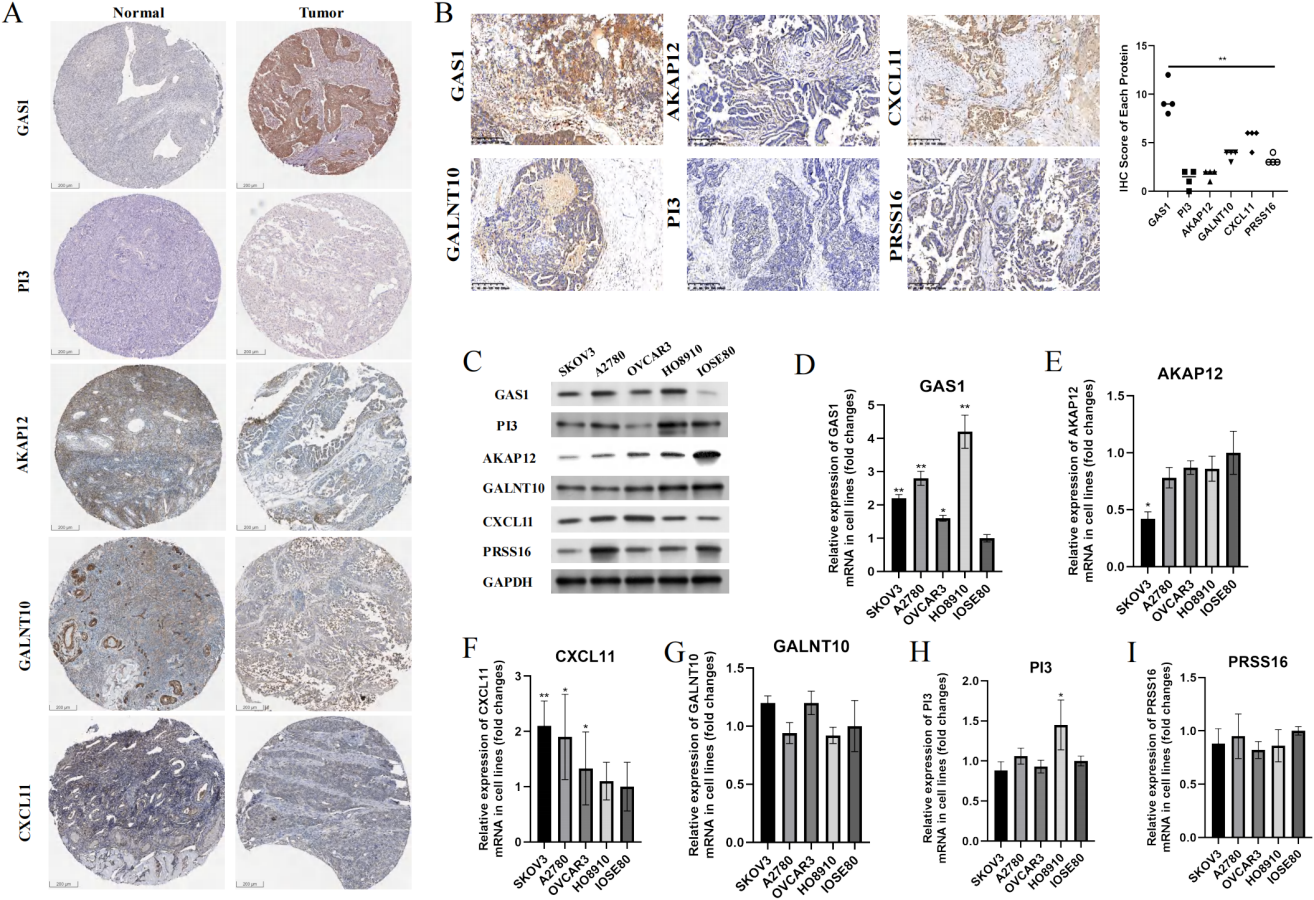
Validation of the expression levels of hub genes in samples. **(A)** IHC results of proteins in RS model from HPA database. **(B)** IHC results of proteins from RS model in our clinical samples. **(C)** Western-blot assay results of proteins from RS model in cell lines. **(D-I)** qPCR results of markers from RS model in cell lines. **p < 0.001.

**Figure 14:**
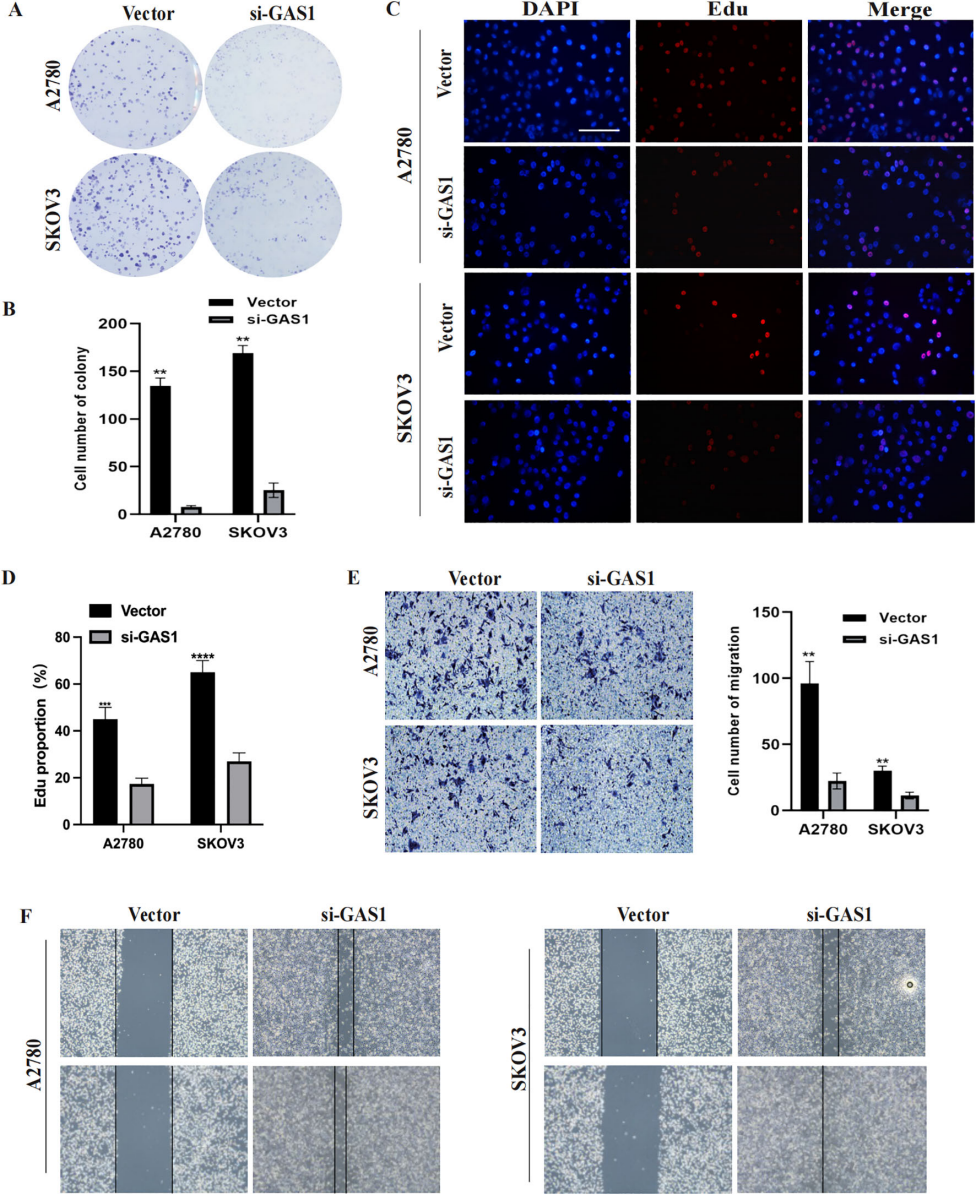
GAS1 regulates OC cell progression. **(A, B)** Colony formation assay results of A2780 and SKOV3 cells transfected with GAS1 siRNA. **C, D)** EdU assay results for A2780 and SKOV3 cells transfected with GAS1 siRNA. **(E)** Transwell assay results of A2780 and SKOV3 cells transfected with GAS1 siRNA.. **(F)** Wound healing assay for A2780 and SKOV3 cells transfected with GAS1 siRNA. **p < 0.001, ****p < 0.0001.

In addition to PRSS16, we explored the protein expression of the other hub genes in the HPA database (https://www.proteinatlas.org/). Among them, GAS1 was significantly overexpressed in OC tissues, whereas AKAP12 and GALNT10 were markedly upregulated in normal tissues. PI3 exhibited low expression in both normal and OC tissues. The remaining proteins were moderately expressed in normal and OC tissues (**Figure 13A**). To further corroborate the results obtained from the HPA database, we conducted validation in OC cell lines and specimens at the protein and mRNA levels through Western blotting and qPCR assays (**Figures 13B–I**), and we discovered that the outcomes aligned with those derived from the HPA database. Due to the coefficient scores, we identified GAS1 as a crucial oncogene from the RS model. Knocking down GAS1 in A2780 and SKOV3 cells resulted in a notable decrease in the proliferative and invasive capacities of OC cells (**Figures 14A–D**). Most notably, diminished EDU fluorescence and weakened reparative abilities were observed following GAS1 knockdown (**Figures 14E, F**).

## 4 Discussion

OC is the most aggressive reproductive system cancer in females worldwide, with a low survival of less than 35% by five years after diagnosis, despite various improved treatment strategies, such as cytoreductive surgery, modified chemotherapy and hormonal therapy (19). Thus, the discovery and development of individualized diagnostic and therapeutic strategies are urgently needed. Fortunately, as medicine has evolved from empirical to evidence-based, personalized, precision medicine has become increasingly achievable. Against the backdrop of the emerging era of bioinformatics, many genetic signatures and corresponding risk models have been mined from internationally available genomic databases and increasingly accepted by the scientific community (20).

Due to the tumor complexity/heterogeneity of neoplasms and their surrounding tumor microenvironment (TME) (5, 21), most OC patients experience recurrence after first-line treatment. Several cells involved in both innate and adaptive immunity, including tumor-associated macrophages (TAMs), tumor-associated neutrophils (TANs), myeloid-derived suppressor cells (MDSCs), γδ T cells, and natural killer (NK) cells, directly or indirectly shape the OC TME (22, 23), which displays unique features leading to immune suppression and tolerance and the impairment of immune system components, including TAMs (24), TANs (25), γδ T cells, and NK cells (26). The immune context of OC acts as a crucial orchestrator of OC progression, playing an indispensable role in predicting patient prognosis.

Cuproptosis, an unconventional cell death mechanism, is involved in numerous pathological conditions, including cancers, albeit mainly through undefined underlying mechanisms. The process of cuproptosis has an unambiguous relationship with mitochondrial respiration (27). Excess copper within cells can be transported to the mitochondria by ionophores and directly bind to lipoylated components of the tricarboxylic acid (TCA) cycle, triggering an accumulation of lipoylated proteins and loss of iron–sulfur cluster proteins, leading to proteotoxic stress and, eventually, cell death (9). However, little work has been performed on optimizing the prognostic model in OC utilizing cuproptosis combined with immune infiltration.

Taken together, in this study, the differential expression of CRGs and TME-related genes was analyzed at the single-cell level, and the correlations between these two types of genes were described, with the active cooperative death of malignant cells further identified. We subsequently identified three molecular subtypes on the basis of differential gene expression between active and inactive cuproptosis cells and demonstrated differential enrichment via multiple analyses. In particular, a prognostic risk model integrating 6 genes was constructed, which was validated with four datasets from major public databases. In addition, the patients were divided into high- and low-risk groups based on the risk model, and the associations between the risk score and multiple features were depicted. Finally, the ability of the risk model to predict the efficacy of immunotherapy and chemotherapy drug resistance was delineated.

Six genes, including PRSS16, CXCL11, PI3, GALNT10, GAS1, and AKAP12, constitute a prognostic risk model, and the RS was calculated. The AUC value of the risk score in the diagnostic ROC analysis using four datasets covering the TCGA and GEO datasets further verified the experimental phenomenon. Among the core genes, PRSS16, encoding a thymus-specific serine protease (TSSP), which is involved in CD4+ T-cell maturation in the thymus, has tumor suppressor activity (28). CXCL11, known as interferon-inducible T-cell alpha chemoattractant (I-TAC), has been reported to be the ligand of specific atypical chemokine receptors, including CXCR7 (29) and GPR182 (30). PI3, encoding elafin, which is a serine protease inhibitor critical for host defense, is reportedly associated with an unfavorable OS and a better immunotherapy response in OC (31). Moreover, GALNT10, an enzyme that mediates protein and lipid modifications, was found to be an independent predictor of prognosis in OC patients with immunosuppression (32). AKAP12, a scaffolding protein, anchors PKA to compartmentalize cycle AMP signaling and was found to be a promoter in tumors (33). Intriguingly, GAS1, which has been reported to play a role in growth suppression, blocks entry into the S phase, prevents the cycling of normal and transformed cells, and functions as a putative tumor suppressor, whereas it had the opposite effect in our study (34). This interplay highlights the complex gene profile heterogeneity of OC, which promotes changes in the tumor microenvironment.

Notably, we investigated the relationship between the risk score and tumor immunology and the differences in tumor immunology among patients with different risk scores. We found that the risk score acts as an independent prognostic factor and is associated with the condition of the tumor microenvironment and the efficacy of immunotherapy. With respect to immunotherapy, a lower risk score was more likely to be beneficial, as it indicated a higher ESTIMATE immune score, fewer mutations in tumor suppressor genes and a greater immune response. In addition, the half-maximal inhibitory concentration (IC50) curves of 138 chemotherapy drugs in the GDSC database were examined to determine their predicted chemotherapeutic effectiveness. Among them, the IC50 values of cyclopamine, ABT-888, and AKT inhibitor were determined. VIII, and ATRA were relatively more common in the high-risk score group, whereas axitinib, BMS.509744, Elesclomol and GNF.2 were relatively more common in the high-risk score groups. Both results were statistically significant after log-rank testing. Accordingly, our prognostic risk model not only predicts the OS rate but is also conducive to more precise therapy choices.

Despite these limitations, including the lack of real-world clinical cohorts and IC50 data from benches, the present study highlights the outstanding ability of the risk model to predict the prognosis of OC and its association with tumor immunology. These findings may contribute to the development of immunotherapy- and chemotherapy-based interventions in the future.

Three studies have confirmed the role of cuproptosis-related genes in OC from the perspectives of molecular subtyping and the risk score. Compared with Li’s results (35), our research focused more on the relationship between the risk score and evaluation indicators such as chemoresistance, genomic mutations, and the efficacy of immunotherapies. We focused primarily on cuproptosis without incorporating ferroptosis, aiming for a more direct exploration of the roles of CRGs in OC. Our study also focused on the role of key genes within the risk score, delving into their oncogenic functions. Unlike Wang’s study (36), our research closely examines the relationships between the risk score and the tumor immune microenvironment as well as immunotherapeutic responses. Additionally, via single-cell analysis, we identified genes that were differentially expressed between the active copper-depleted malignant cell population and the inactive cell population to characterize the molecular subtypes in the TCGA ovarian cancer cohort. Finally, compared with Zhang’s research (8), our strength lies in uncovering the oncogenic functions of key genes within the risk score and validating these findings in samples. Furthermore, our molecular subtyping method is more specific and precise.

In conclusion, our study provides a novel risk score model including 6 hub genes associated with cuproptosis and immune infiltration to predict OC prognosis as well as clinical efficacy.

## Data Availability

The original contributions presented in the study are included in the article/**Supplementary Material**. Further inquiries can be directed to the corresponding authors.
