# Supplementary material for: Integrated multiomics characterization reveals cuproptosis-related hub genes for predicting the prognosis and clinical efficacy of ovarian cancer
Source: Front Immunol. 2024 Nov 12;15:1452294. doi: 10.3389/fimmu.2024.1452294 (PMC11588705; doi:10.3389/fimmu.2024.1452294)
Supplement: Supplementary file 1 [file DataSheet1.docx]

**Figure S1. Enrichment Analysis of DEGs among TCGA Molecular Subtypes.** A: Box plot showing the expression differences of 23 immune checkpoint inhibitors between molecular subtypes; B: Bubble plot of KEGG enrichment analysis for differentially expressed genes between subtypes, where the size of the dots represents the number of enriched marker genes and the color represents the significance of enrichment; C-E: Bubble plots of GO functional enrichment analysis for differentially expressed genes between subtypes, including GOBP (Gene Ontology Biological Process), GOMF (Gene Ontology Molecular Function), and GOCC (Gene Ontology Cellular Component).

**Figure S2. The different cuproptosis expression patterns of the three subtypes.** A: Significant expression result of CRGs in the three subtypes. B: Heatmap result of CRGs in the three subtypes. C: ssGSEA of CRGs score in the three subtypes.

**Figure S3.Validation of the model's prognostic efficacy using the TCGA testing dataset.** A-C: Ternary plot of risk in the TCGA test set, comprising a heatmap illustrating model gene expression within the model's stratification, a scatter plot depicting risk scores, and a scatter plot illustrating survival time; D: KM curve for the TCGA test set; E: ROC curve for the TCGA test set.

**Figure S4.Validation of the model's prognostic efficacy using the TCGA overall dataset.** A-C: Ternary plot of risk in the TCGA test set, comprising a heatmap illustrating model gene expression within the model's stratification, a scatter plot depicting risk scores, and a scatter plot illustrating survival time; D: KM curve for the TCGA test set; E: ROC curve for the TCGA test set.

**Figure S5. Validation of the model's prognostic efficacy using GSE26712 dataset.** A-C: Ternary plot of risk in the GSE26712 dataset, comprising a heatmap illustrating model gene expression within the model's stratification, a scatter plot depicting risk scores, and a scatter plot illustrating survival time. Red represents the high-risk group, while green represents the low-risk group; D-E: KM curve and ROC curve for the GSE26712 dataset.

**Figure S6. Differences of HALLMARK enrichment in Riskscore Model.** A: Discrepancies in HALLMARK pathway enrichment scores between high and low-risk groups, with asterisks denoting significance of the differences. B: Correlation heatmap illustrating the association between model gene expression, risk assessment, and HALLMARK pathway enrichment scores. The depth of color signifies the strength of the correlation, while asterisks indicate significance. C-F: Scatter plots depicting the correlation between enrichment scores of four immunological gene sets and risk assessments. The absolute value of R reflects the strength of the correlation, with positive values indicating a positive correlation, negative values indicating a negative correlation, and p-values representing significance.
