# Supplementary material for: Integrated multiomics characterization reveals cuproptosis-related hub genes for predicting the prognosis and clinical efficacy of ovarian cancer
Source: Front Immunol. 2024 Nov 12;15:1452294. doi: 10.3389/fimmu.2024.1452294 (PMC11588705; doi:10.3389/fimmu.2024.1452294)
Supplement: Supplementary file 2 [file Image1.pdf]

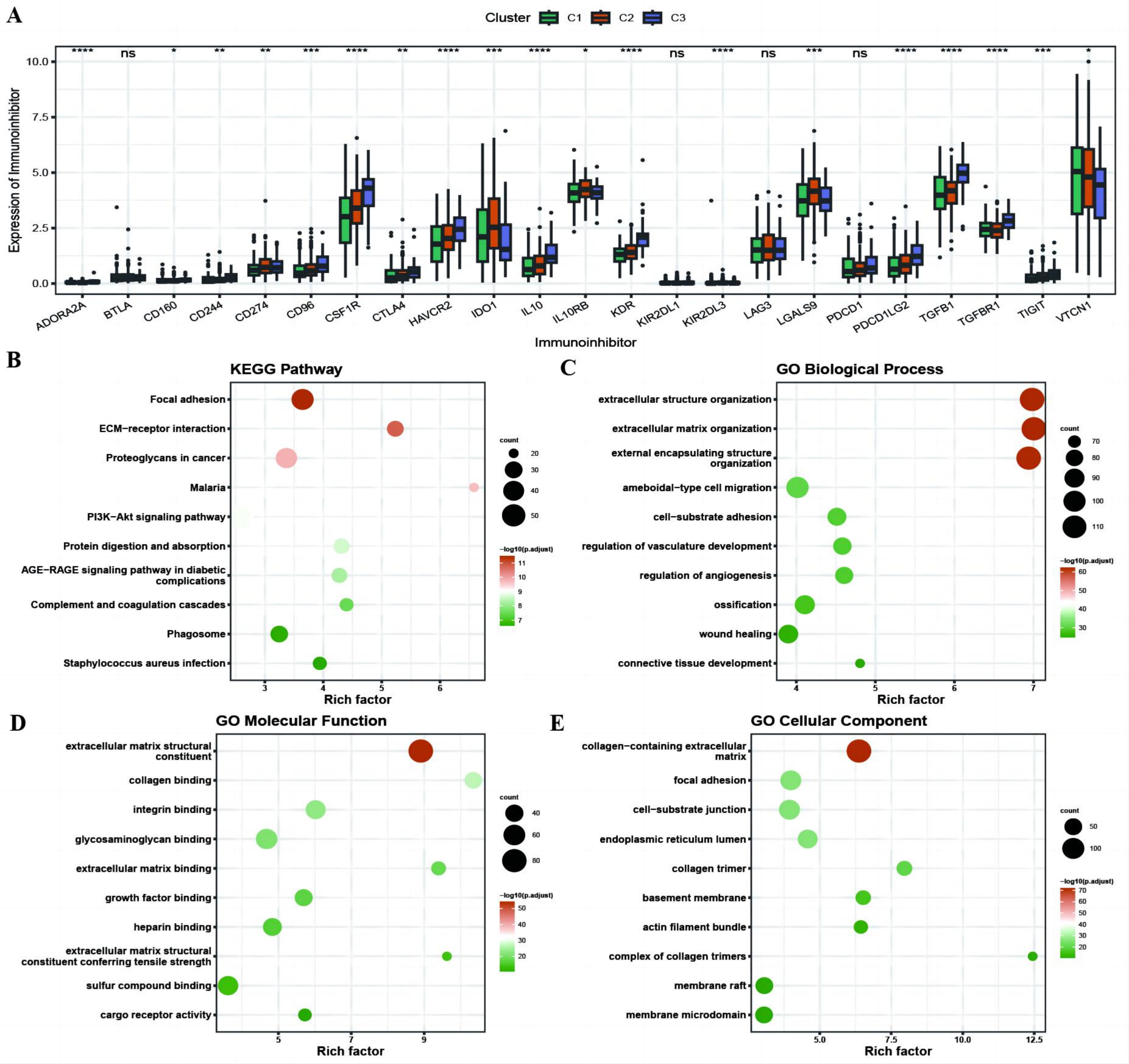
A
Cluster C1 C2 C3
Expression of Immunoinhibitor
Immunoinhibitor
**** ns * ** ** *** **** ** **** *** **** * **** ns **** ns *** ns **** **** **** *** *
ADORA2A BTLA CD160 CD244 CD274 CD96 CSF1R CTLA4 HAVCR2 IDO1 IL10 IL10RB KDR KIR2DL1 KIR2DL3 LAG3 LGALS9 PDCD1 PDCD1LG2 TGFB1 TGFBR1 TIGIT VTCN1
B
KEGG Pathway
Focal adhesion
ECM−receptor interaction
Proteoglycans in cancer
Malaria
PI3K−Akt signaling pathway
Protein digestion and absorption
AGE−RAGE signaling pathway in diabetic complications
Complement and coagulation cascades
Phagosome
Staphylococcus aureus infection
Rich factor
count
−log10(p.adjust)
C
GO Biological Process
extracellular structure organization
extracellular matrix organization
external encapsulating structure organization
ameboidal−type cell migration
cell−substrate adhesion
regulation of vasculature development
regulation of angiogenesis
ossification
wound healing
connective tissue development
Rich factor
count
−log10(p.adjust)
D
GO Molecular Function
extracellular matrix structural constituent
collagen binding
integrin binding
glycosaminoglycan binding
extracellular matrix binding
growth factor binding
heparin binding
extracellular matrix structural constituent conferring tensile strength
sulfur compound binding
cargo receptor activity
Rich factor
count
−log10(p.adjust)
E
GO Cellular Component
collagen−containing extracellular matrix
focal adhesion
cell−substrate junction
endoplasmic reticulum lumen
collagen trimer
basement membrane
actin filament bundle
complex of collagen trimers
membrane raft
membrane microdomain
Rich factor
count
−log10(p.adjust)

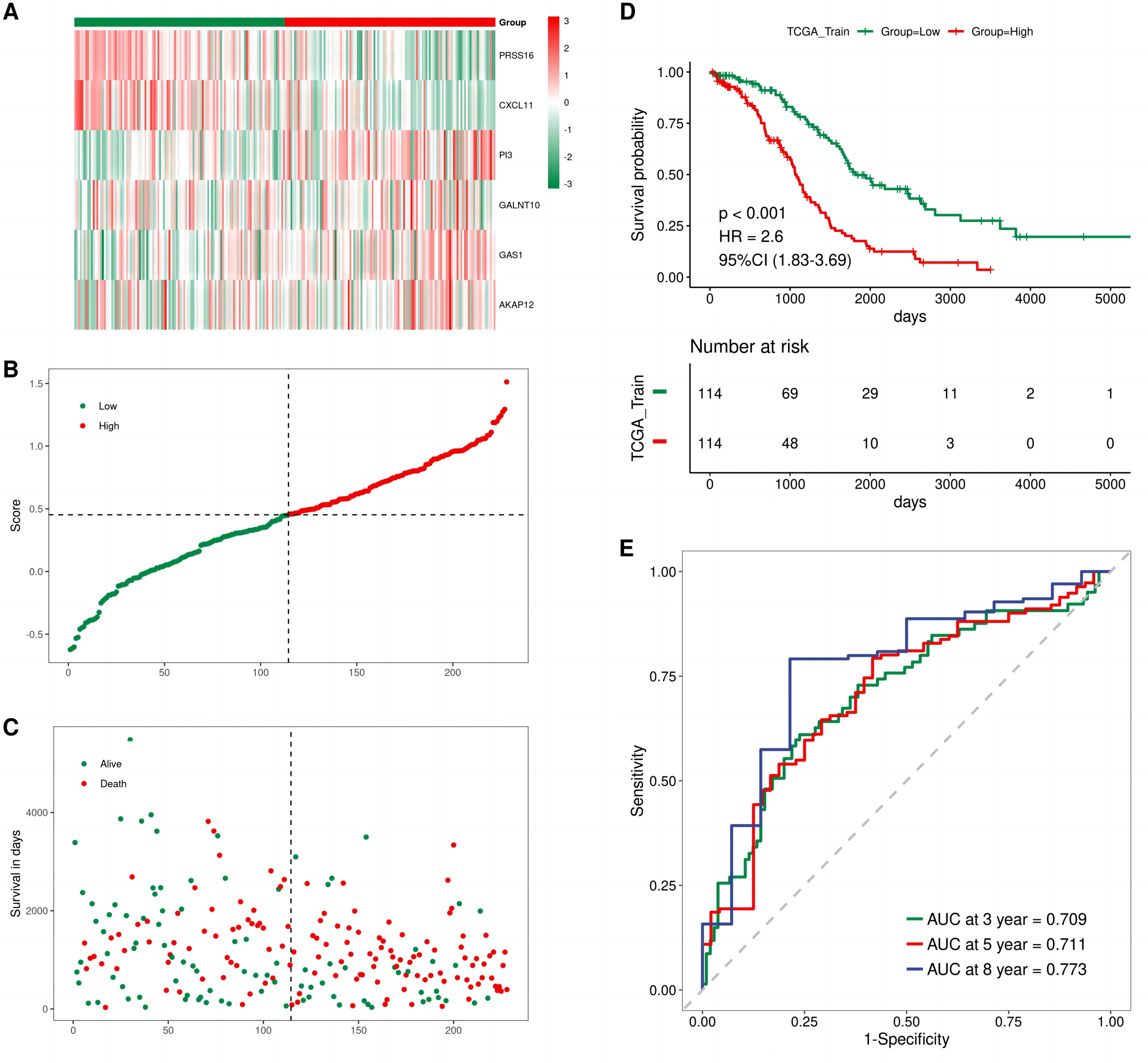

A
Group
PRSS16
CXCL11
PI3
GALNT10
GAS1
AKAP12
B
Low
High
Score
C
Alive
Death
Survival in days
D
TCGA_Train
Group=Low
Group=High
Survival probability
p < 0.001
HR = 2.6
95%CI (1.83-3.69)
days
Number at risk
114 69 29 11 2 1
114 48 10 3 0 0
E
Sensitivity
1-Specificity
AUC at 3 year = 0.709
AUC at 5 year = 0.711
AUC at 8 year = 0.773

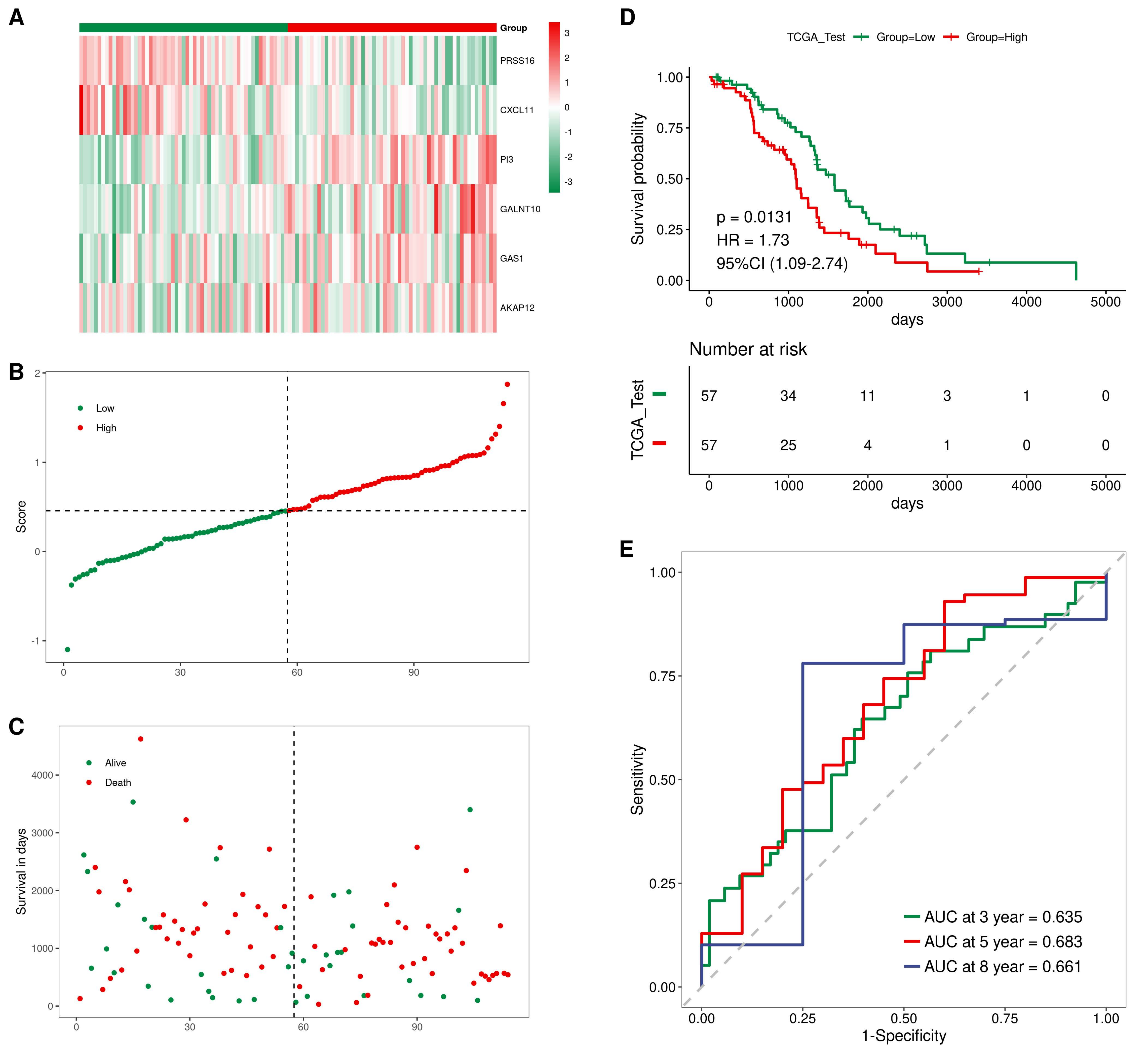
A
Group
PRSS16
CXCL11
PI3
GALNT10
GAS1
AKAP12
B
Low
High
Score
C
Alive
Death
Survival in days
D
TCGA_Test
Group=Low
Group=High
Survival probability
p = 0.0131
HR = 1.73
95%CI (1.09-2.74)
days
Number at risk
TCGA_Test
57 34 11 3 1 0
57 25 4 1 0 0
days
E
Sensitivity
1-Specificity
AUC at 3 year = 0.635
AUC at 5 year = 0.683
AUC at 8 year = 0.661

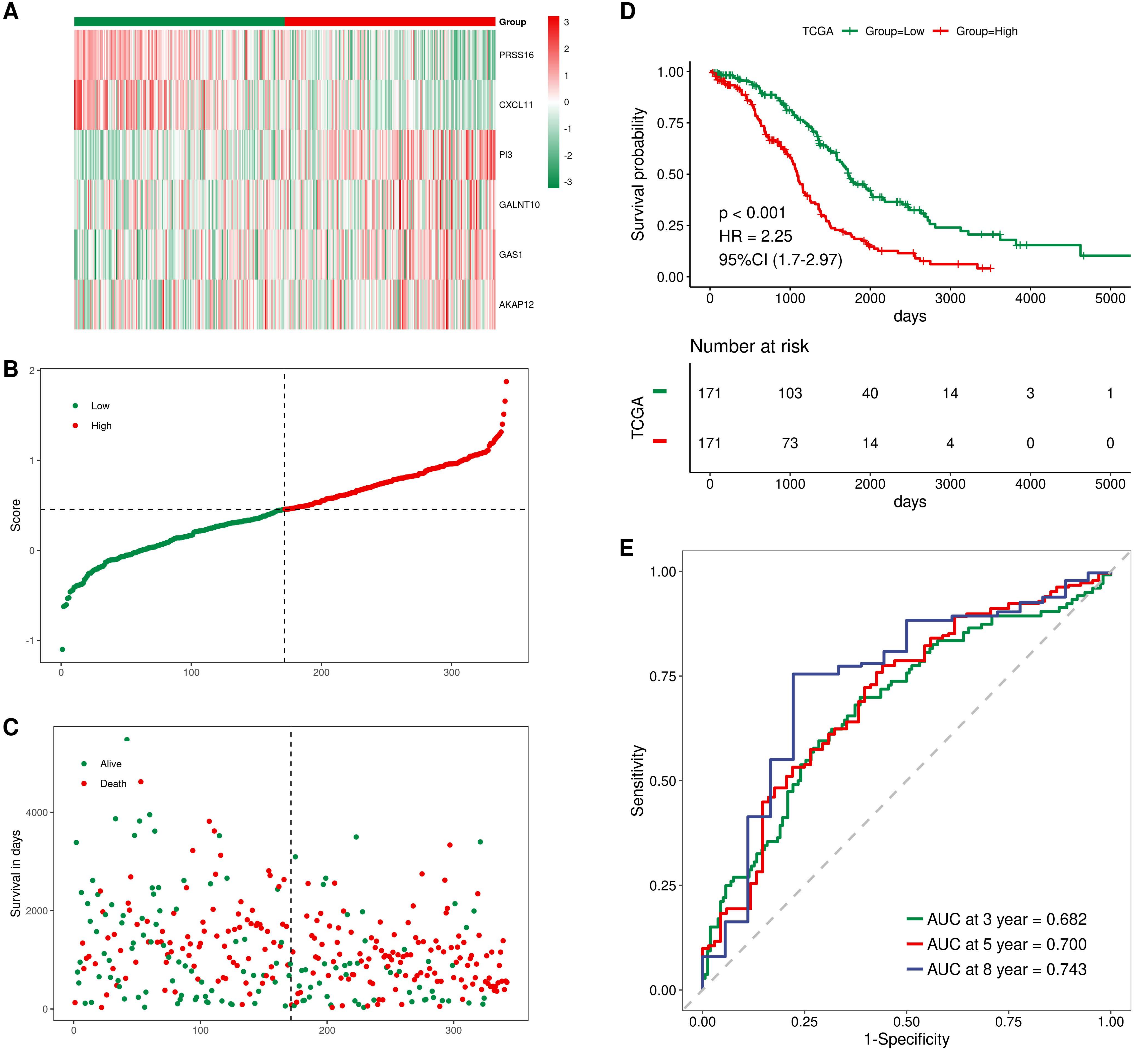

A
Group
PRSS16
CXCL11
PI3
GALNT10
GAS1
AKAP12
B
Low
High
Score
C
Alive
Death
Survival in days
D
TCGA
Group=Low
Group=High
Survival probability
p < 0.001
HR = 2.25
95%CI (1.7-2.97)
days
Number at risk
TCGA
171 103 40 14 3 1
171 73 14 4 0 0
days
E
Sensitivity
1-Specificity
AUC at 3 year = 0.682
AUC at 5 year = 0.700
AUC at 8 year = 0.743

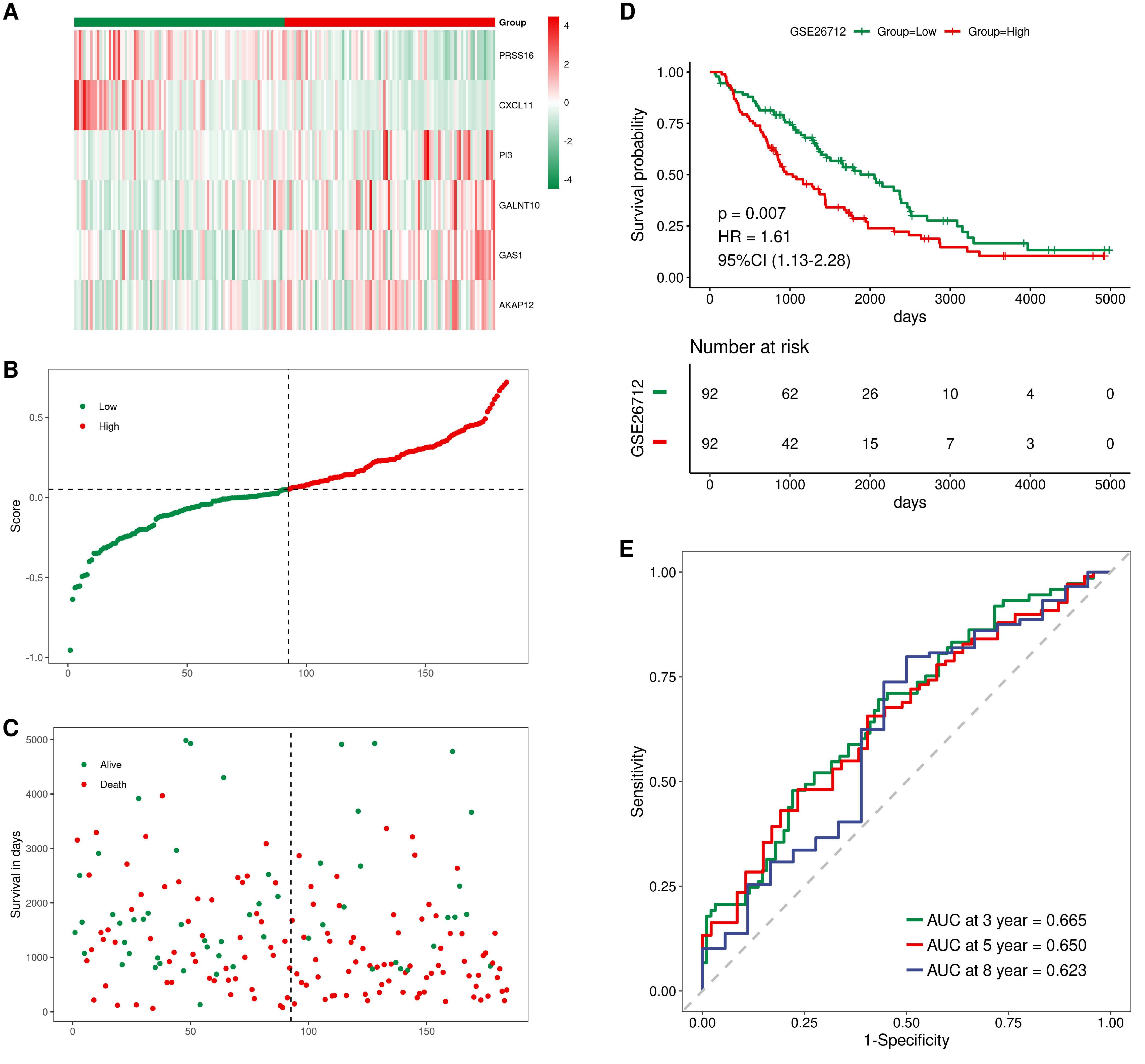
A
Group
PRSS16
CXCL11
PI3
GALNT10
GAS1
AKAP12
4
2
0
-2
-4
B
Low
High
Score
0.5
0.0
-0.5
-1.0
0
50
100
150
C
Alive
Death
Survival in days
5000
4000
3000
2000
1000
0
0
50
100
150
D
GSE26712
Group=Low
Group=High
Survival probability
1.00
0.75
0.50
0.25
0.00
p = 0.007
HR = 1.61
95%CI (1.13-2.28)
0
1000
2000
3000
4000
5000
days
Number at risk
GSE26712
92 62 26 10 4 0
92 42 15 7 3 0
0
1000
2000
3000
4000
5000
days
E
Sensitivity
1.00
0.75
0.50
0.25
0.00
AUC at 3 year = 0.665
AUC at 5 year = 0.650
AUC at 8 year = 0.623
0.00
0.25
0.50
0.75
1.00
1-Specificity

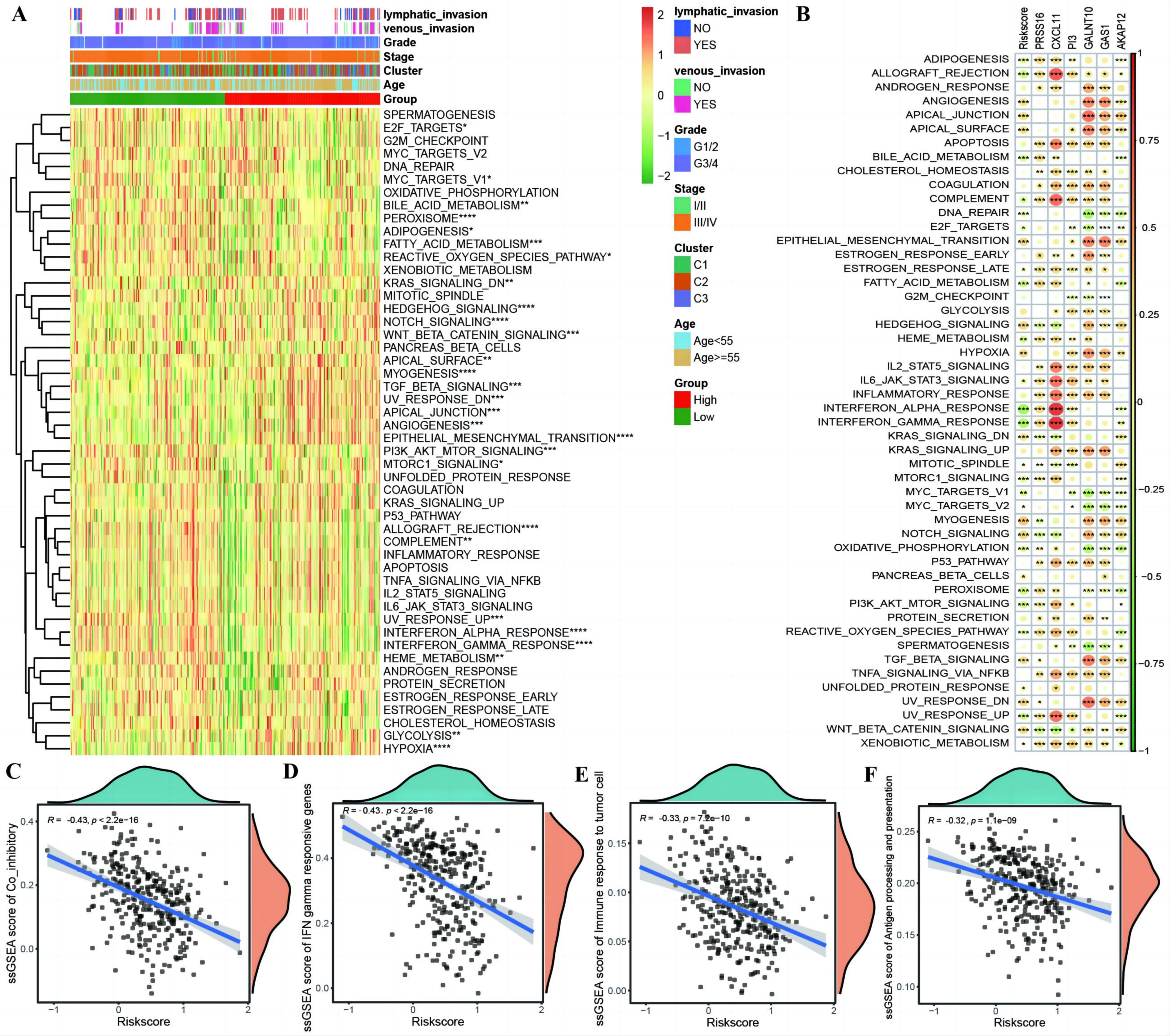
A
lymphatic_invasion
venous_invasion
Grade
Stage
Cluster
Age
Group
SPERMATOGENESIS
E2F_TARGETS*
G2M_CHECKPOINT
MYC_TARGETS_V2
DNA_REPAIR
MYC_TARGETS_V1*
OXIDATIVE_PHOSPHORYLATION
BILE_ACID_METABOLISM**
PEROXISOME****
ADIPOGENESIS*
FATTY_ACID_METABOLISM***
REACTIVE_OXYGEN_SPECIES_PATHWAY*
XENOBIOTIC_METABOLISM
KRAS_SIGNALING_DN**
MITOTIC_SPINDLE
HEDGEHOG_SIGNALING****
NOTCH_SIGNALING****
WNT_BETA_CATENIN_SIGNALING***
PANCREAS_BETA_CELLS
APICAL_SURFACE**
MYOGENESIS****
TGF_BETA_SIGNALING***
UV_RESPONSE_DN***
APICAL_JUNCTION***
ANGIOGENESIS***
EPITHELIAL_MESENCHYMAL_TRANSITION****
PI3K_AKT_MTOR_SIGNALING***
MTORC1_SIGNALING*
UNFOLDED_PROTEIN_RESPONSE
COAGULATION
KRAS_SIGNALING_UP
P53_PATHWAY
ALLOGRAFT_REJECTION****
COMPLEMENT**
INFLAMMATORY_RESPONSE
APOPTOSIS
TNFA_SIGNALING_VIA_NFKB
IL2_STAT5_SIGNALING
IL6_JAK_STAT3_SIGNALING
UV_RESPONSE_UP***
INTERFERON_ALPHA_RESPONSE****
INTERFERON_GAMMA_RESPONSE****
HEME_METABOLISM**
ANDROGEN_RESPONSE
PROTEIN_SECRETION
ESTROGEN_RESPONSE_EARLY
ESTROGEN_RESPONSE_LATE
CHOLESTEROL_HOMEOSTASIS
GLYCOLYSIS**
HYPOXIA****
2 1 0 −1 −2
lymphatic_invasion: NO, YES
venous_invasion: NO, YES
Grade: G1/2, G3/4
Stage: I/II, III/IV
Cluster: C1, C2, C3
Age: Age<55, Age>=55
Group: High, Low
B
Riskscore PRSS16 CXCL11 PI3 GALNT10 GAS1 AKAP12
ADIPOGENESIS
ALLOGRAFT_REJECTION
ANDROGEN_RESPONSE
ANGIOGENESIS
APICAL_JUNCTION
APICAL_SURFACE
APOPTOSIS
BILE_ACID_METABOLISM
CHOLESTEROL_HOMEOSTASIS
COAGULATION
COMPLEMENT
DNA_REPAIR
E2F_TARGETS
EPITHELIAL_MESENCHYMAL_TRANSITION
ESTROGEN_RESPONSE_EARLY
ESTROGEN_RESPONSE_LATE
FATTY_ACID_METABOLISM
G2M_CHECKPOINT
GLYCOLYSIS
HEDGEHOG_SIGNALING
HEME_METABOLISM
HYPOXIA
IL2_STAT5_SIGNALING
IL6_JAK_STAT3_SIGNALING
INFLAMMATORY_RESPONSE
INTERFERON_ALPHA_RESPONSE
INTERFERON_GAMMA_RESPONSE
KRAS_SIGNALING_DN
KRAS_SIGNALING_UP
MITOTIC_SPINDLE
MTORC1_SIGNALING
MYC_TARGETS_V1
MYC_TARGETS_V2
MYOGENESIS
NOTCH_SIGNALING
OXIDATIVE_PHOSPHORYLATION
P53_PATHWAY
PANCREAS_BETA_CELLS
PEROXISOME
PI3K_AKT_MTOR_SIGNALING
PROTEIN_SECRETION
REACTIVE_OXYGEN_SPECIES_PATHWAY
SPERMATOGENESIS
TGF_BETA_SIGNALING
TNFA_SIGNALING_VIA_NFKB
UNFOLDED_PROTEIN_RESPONSE
UV_RESPONSE_DN
UV_RESPONSE_UP
WNT_BETA_CATENIN_SIGNALING
XENOBIOTIC_METABOLISM
1 0.75 0.5 0.25 0 −0.25 −0.5 −0.75 −1
C
R = -0.43, p < 2.2e−16
ssGSEA score of Co_inhibitory
Riskscore
D
R = -0.43, p < 2.2e−16
ssGSEA score of IFN gamma responsive genes
Riskscore
E
R = -0.33, p = 7.2e−10
ssGSEA score of Immune response to tumor cell
Riskscore
F
R = -0.32, p = 1.1e−09
ssGSEA score of Antigen processing and presentation
Riskscore
